# NKp30 and NKG2D contribute to natural killer cell-mediated recognition of HIV-infected cells

**DOI:** 10.1016/j.isci.2025.113548

**Published:** 2025-09-12

**Authors:** Ruoxi Pi, Nancy Q. Zhao, Allison J. Bien, Thanmayi Ranganath, Christof Seiler, Susan Holmes, Alexander Marson, David N. Nguyen, Catherine A. Blish

**Affiliations:** 1 Department of Medicine, Division of Infectious Diseases and Geographic Medicine, Stanford University, Stanford, CA, USA; 2 Immunology Program, Stanford University, Stanford, CA, USA; 3 Innovative Genomics Institute, University of California, Berkeley, Berkeley, CA, USA; 4 Department of Molecular and Cell Biology, Division of Immunology & Molecular Medicine, University of California, Berkeley, Berkeley, CA, USA; 5 Department of Advanced Computing Sciences, Maastricht University, Maastricht, the Netherlands; 6 Mathematics Centre Maastricht, Maastricht University, Maastricht, the Netherlands; 7 Center of Experimental Rheumatology, Department of Rheumatology, University Hospital Zurich, University of Zurich, Zurich, Switzerland; 8 Department of Statistics, Stanford University, Stanford, CA, USA; 9 Department of Medicine, Division of Infectious Diseases, University of California, San Francisco, San Francisco, CA, USA; 10 Department of Microbiology and Immunology, University of California, San Francisco, San Francisco, CA, USA; 11 Parker Institute for Cancer Immunotherapy, San Francisco, CA, USA; 12 Helen Diller Family Comprehensive Cancer Center, University of California, San Francisco, San Francisco, CA, USA; 13 Gladstone Institutes, San Francisco, CA, USA; 14 Chan Zuckerberg Biohub, San Francisco, CA, USA

**Keywords:** Natural sciences, Biological sciences, Immunology, Immunity

## Abstract

Natural killer (NK) cells respond rapidly in early HIV-1 infection. HIV-1 prevention and control strategies harnessing NK cells could be enabled by mechanistic understanding of how NK cells recognize HIV-infected T cells. Here, we profiled the phenotype of human primary NK cells responsive to autologous newly HIV-infected CD4 T cells *in vitro*. We characterized the patterns of NK cell ligand expression on CD4 T cells at baseline and after infection with a panel of transmitted/founder HIV-1 strains to identify key receptor-ligand pairings. CRISPR editing of CD4 T cells to knock out the NKp30 ligand B7-H6, or the NKG2D ligand MICB reduced NK cell responses to HIV-infected cells in some donors. Blockade of NKp30 or NKG2D on NK cells compromised their specificity of killing HIV-infected cells. Collectively, we identified receptor-ligand pairs including NKp30:B7-H6 and NKG2D:MICB that contribute to NK cell recognition of HIV-infected cells.

## Highlights

• Ligands of NKp30 and NKG2D are upregulated on HIV-infected CD4 T cells
• Ligand expression varies more between individuals than between viral strains
• NKp30 and NKG2D facilitate HIV-specific cytotoxicity mediated by NK cells

## Graphical abstract

**Figure:**
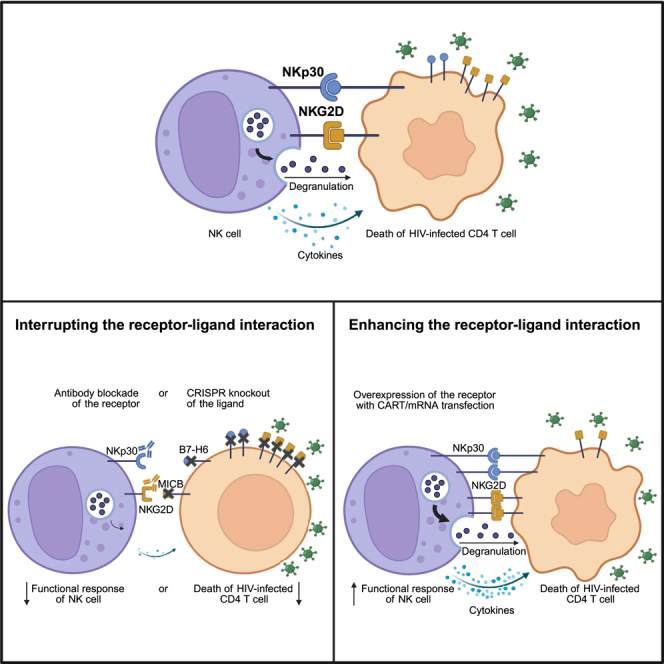

## Introduction

Human immunodeficiency virus (HIV) remains a significant global public health issue, with 40.8 million people still living with HIV in 2024 and an estimated 1.3 million new infections yearly highlighting the need for improved strategies to prevent infection.^1^ Natural killer (NK) cells are innate immune cells that are among the earliest responders to viral infection. They rapidly expand during the earliest stages of HIV infection,^2^^,^^3^ and have been implicated in both acquisition and immune control of HIV. Highly exposed seronegative individuals have increased constitutive NK cell activity,^4^^,^^5^ suggesting that NK cell activity can protect against infection. Further, the number of CXCR5^+^ NK cells in B cell follicles of secondary lymphoid tissue (SLT) inversely correlated with the viral load of HIV, simian immunodeficiency virus (SIV), or SHIV/HIV hybrid virus (SHIV) during chronic infections.^6^^,^^7^^,^^8^ Higher functionality of CXCR5^+^ NK cells indicated by CD107a or IFNγ expression compared to CXCR5^-^ counterparts suggests that NK cells may limit virus spread in T follicular helper cells (T_FH_), one of the cell types that naturally harbor persistent reservoirs of latent HIV.^6^^,^^7^^,^^8^ Finally, depletion of NK cells led to elevated HIV viral load in plasma, lymph nodes, the spleen and liver in humanized mice, indicating the role of NK cells in suppressing HIV infection.^9^ Improved understanding of the mechanisms NK cells utilize to recognize and respond to HIV-1 infection could allow these immune functions to be harnessed to prevent, control, or even cure infection.

Much effort on NK cell recognition mechanisms have focused on interactions between HLA class I molecules on HIV-infected cells and the killer immunoglobulin receptors (KIRs) on NK cells. This is particularly relevant as multiple studies have demonstrated the importance of the KIR3DL1–HLA-B allotype interaction in slower disease progression to AIDS and lower viral load,^10^ reduced risk of HIV infection in HIV-exposed seronegative subjects,^11^ and improved control of HIV replication *in vitro*.^12^^,^^13^ HIV-1 infection induces downregulation of expression of the classical HLA class I molecules HLA-A, HLA-B, and HLA-C on infected cells,^14^^,^^15^^,^^16^ which may be of help in the evasion of cytotoxic T lymphocyte responses but can instead trigger recognition by educated NK cells. Consistent with the idea that educated NK cells can play a critical role in HIV-1 control, high expression of both surface KIR3DL1 and its HLA ligand (the Bw4 epitope) leads to the generation of more potently reactive NK cells to HIV-infected cells,^17^ presumably via stronger education and hence reactivity to “missing self”. In addition, NK cell education via KIRs determines their ability to respond to HIV-mediated downregulation of their cognate HLA class I ligand on target cells *in vitro*.^18^

Besides the interaction between HLA class I molecules and KIRs, there is growing evidence that HLA-E, a nonclassical HLA class I molecule, which interacts with NK cell receptors CD94/NKG2A and CD94/NKG2C, can regulate NK cell function in response to HIV-infected cells. Multiple HIV-derived peptides, when presented by HLA-E, have been identified to induce elevated functionality of NK cells, including enhanced cytotoxicity, degranulation or TNF-α secretion.^19^^,^^20^^,^^21^ It has been hypothesized that peptides binding to HLA-E can either interrupt the inhibitory interaction between HLA-E and CD94/NKG2A, or facilitate NK cell activation mediated by CD94/NKG2C. Nef of multiple HIV strains downregulates HLA-E in primary CD4 T cells,^22^ potentially influencing peptide presentation by HLA-E and interaction between HLA-E and its receptors.

In addition to recognition of HLA class I molecules, infected cells can also modulate the expression of ligands for other NK cell receptors, mediated mostly by HIV-1 accessory proteins. For example, Vpr is known to upregulate NKG2D ligands, particularly ULBP2.^23^ Conversely, Vpu and Nef downregulate these ligands, providing an avenue for immune escape.^24^ Vpu also downregulates NTB-A, a homotypic co-activating signaling lymphocytic activation molecule (SLAM) family receptor found on both CD4 T cells as well as NK cells, and this has been reported to prevent NK cell activation against infected cells.^24^^,^^25^^,^^26^ Vpu of multiple HIV strains downregulates CD48, the ligand of another SLAM family receptor, CD244.^26^ Impairment of NTB-A-NTB-A and CD48-CD244 interactions dampens antibody-dependent cellular cytotoxicity (ADCC) toward HIV-infected primary CD4 T cells.^26^ HIV-2 infection induces the upregulation of B7-H6, which may cause downmodulation of NKp30 on NK cells through persistent interaction.^27^ However, as NK cells integrate signaling from all their surface receptors to determine activation, it remains unclear whether the overall ligand modulation on infected cells leads to NK cell susceptibility or escape, and which receptor-ligand interactions are most prominently involved. The recent use of high-dimensional mass cytometry has allowed the simultaneous profiling of the numerous NK cell surface receptors and their respective ligands on infected cells,^28^^,^^29^ providing a powerful tool to address overall pathogen-driven patterns of ligand modulation.

While the traditional paradigm suggests that innate immune cells are activated by invariant pathogen signatures and do not possess specificity, recent work has shown that this is not always the case. Indeed, a pandemic influenza H1N1 strain differentially modulates NK cell ligands and triggers different levels of IFN-ɣ production by NK cells than a co-circulating H3N2 strain.^30^ As such, variability between viral strains that leads to different pathways of NK cell ligand modulation may lead to strain-specific responses by the innate immune system. HIV is extremely genetically diverse—even more so than influenza^31^—and hence it remains unclear if particular strains may have different patterns of ligand modulation or induce differential recognition by NK cells.

Here, we use mass cytometry to simultaneously profile the expression of known NK cell ligands on HIV-infected CD4 T cells, as well as the phenotypes of NK cells responding to autologous HIV-infected cells. We analyzed NK cell ligand expression on CD4 T cells infected with a panel of HIV strains comprising transmitted/founder (T/F) and laboratory-adapted subtype B strains and an early subtype A strain to determine if patterns of ligand expression were differentially modulated by different strains. We used these analyses to determine a set of candidate receptor-ligand interactions that mediate NK cell recognition of HIV infection. Knockout of these T cell surface ligands individually with CRISPR editing, and comparison between NK cell response to HIV-infected and to mock-infected CD4 T cells, suggested a potential role for B7-H6 (a ligand for NKp30) and major histocompatibility complex (MHC) class I chain-related protein B (MICB, a NKG2D ligand) in HIV-specific recognition by NK cells. We overexpressed NKp30 and NKG2D with mRNA transfection and confirmed their role of mediating NK cell recognition of HIV-infected and mock-infected CD4 T cells. Blockade of NKp30 or NKG2D with antibodies compromised the specificity of NK cell cytotoxicity on HIV-infected CD4 T cells.

## Results

### An *in vitro* autologous co-culture system allows the detection of NK cell responses against HIV

**Figure 1:**
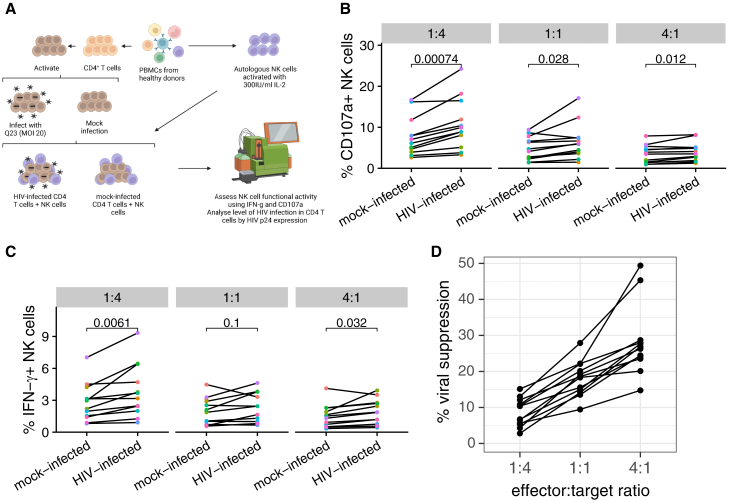
An *in vitro* NK-CD4 T cell co-culture system allows the dissection of responses to HIV-infected cells An *in vitro* co-culture system was used to detect NK cell responses to autologous CD4 T cells infected with HIV strain Q23 or mock-infected. For HIV-infected CD4 T cells, effector-to-target ratios were calculated based on the total number of Gag p24^+^ and bystander cells in the CD4 T cells that were inoculated with HIV. (A) Schematic demonstrating the design of the co-culture system. This image was created with BioRender.com. (B–D) Summary data for NK cell responses, for (B) cytolytic degranulation by the marker CD107a, (C) IFN-ɣ production, and (D) viral suppression, at varying E:T ratios for healthy donors tested in this assay. Viral suppression was calculated as the percent decrease in CD4 T cells positive for the HIV Gag antigen p24 in the presence vs. absence of NK cells. Statistical analysis in (B) and (C) were performed with paired t test (n = 12).

We first optimized an *in vitro* co-culture system to allow the detection of HIV-specific responses, using CD4 T cells infected *in vitro* with the subtype A HIV-1 virus Q23-17 (shortened as Q23) co-cultured with autologous NK cells.^32^ We stimulated primary CD4 T cells derived from peripheral blood mononuclear cells (PBMCs) of healthy donors using plate-bound anti-CD3 antibody, anti-CD28/CD49d antibodies, and phytohemagglutinin-L (PHA-L) for 48 h, followed by magnetofection with HIV (Figure 1A). In comparison to the laboratory-adapted strain NL4-3 (multiplicity of infection (MOI) of 0.5), which resulted in the highest percentage of Gag p24^+^ cells and significant downregulation of CD4 in Gag p24^+^ cells at 48 h post-infection (hpi), we observed that the percentage of Gag p24^+^ cells after Q23 infection (MOI of 10) declined from 24 hpi to 48 hpi and 72 hpi in most donors, with limited downregulation of CD4 (Figures S1A–S1D). Treatment with raltegravir, an HIV integrase inhibitor, caused decrease in the percentage of Gag p24^+^CD4^−^ cells at 48 and 72 hpi with NL4-3, likely because integration is required for the expression of viral accessory proteins that downregulate CD4 in infected cells (Figure S1G).^33^^,^^34^^,^^35^ Raltegravir treatment also caused reduction in the percentage of Gag p24^+^CD4^−^ cells in total CD4 T cells at 48 and 72 hpi with Q23; however, it did not considerably influence the percentage of Gag p24^+^CD4^+^ in total CD4 T cells (Figures S1E and S1F), suggesting that the majority of Gag p24^+^ cells were pre-integration events. The presence of Gag p24^+^CD4^+^ population is possibly due to the synchronized infection achieved through magnetofection, which allows simultaneous entry of a large amount of viral particles with detectable Gag staining by flow cytometry.^36^ We isolated autologous total NK cells from PBMCs (Figure S1H) and stimulated them with interleukin-2 (IL-2) for 72 h. Stimulation with IL-2 enhances the expression of functional markers including perforin, granzyme B, and MIP-1β in NK cells, which potentially elevates NK cell responses after the recognition of target cells.^37^ We co-cultured the CD4 T cells that have been inoculated with Q23 at 24 hpi and mock-infected CD4 T cells (not inoculated with HIV but treated in parallel otherwise) with IL-2 stimulated NK cells at various E:T ratios for 4 h (Figure 1A), and observed consistent NK cell responses with both cytolytic degranulation (by the expression of the degranulation marker CD107a; Figure 1B) as well as cytokine production (for the canonical NK cell cytokine IFN-ɣ; Figure 1C). Notably, healthy donors differed considerably in their functional response to autologous, HIV-infected CD4 T cells. Both degranulation and cytokine production scaled negatively with increasing effector:target (E:T) ratio. In addition, we observed effective viral suppression mediated by NK cells with a reduction in levels of p24^+^ cells in the presence of NK cells; viral suppression scaled positively with E:T ratio, as expected (Figure 1D). As such, we were able to further utilize this *in vitro* system to probe-specific interactions involved in NK cell recognition of HIV and the initiation of downstream HIV-specific responses.

### Phenotypic profiling of HIV-responsive NK cells with mass cytometry

To determine the receptors involved in triggering the NK cell functional responses observed, we used mass cytometry to simultaneously profile expression of NK cell surface receptors and functional markers in our co-culture system, with mock- and HIV-infected cells. NK cells were identified by gating on CD3^−^ cells that expressed either CD56 or CD16. We used the uniform manifold approximation and projection (UMAP) algorithm to visualize these NK cells from co-cultures with mock- or HIV-infected CD4 T cells. We observed the two canonical NK cell subsets by the expression of the two lineage markers, CD56^bright^ or CD16^+^. To identify cells involved in anti-HIV responses, we reasoned that cells performing any of the canonical NK cell functions, including cytotoxic activity (indirectly measured by CD107a induction) and cytokine secretion (IFN-ɣ and TNF-ɑ) could contribute to the suppression of HIV-1 replication in infected CD4 T cells. We hence visualized the expression of functional markers CD107a, IFN-ɣ and TNF to try to identify regions of HIV-responsive NK cells, but found that functionally active cells were not closely associated into a discrete phenotypic cluster (Figure S2A).

**Figure 2:**
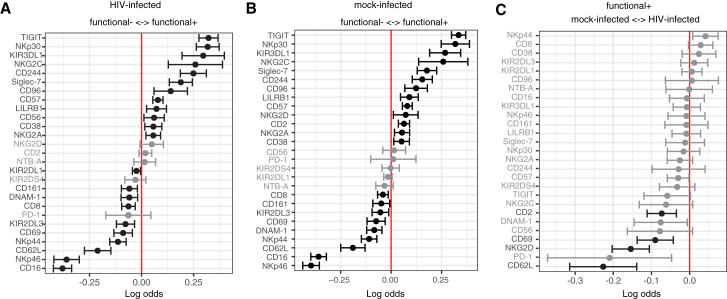
Mass cytometry profiling of NK cell receptors and functional markers identifies the phenotypic features of responding NK cells (A and B) A generalized linear mixed model (GLMM) with paired comparison was used to compare non-responding (functional^−^) and responding (functional^+^) NK cells, after NK cells were co-cultured with HIV-infected (A) or mock-infected (B) CD4 T cells from the same individual (*n* = 20) at effector:target ratio of 1:4. (C) A GLMM with paired comparison was used to compare responding cells from NK cells in co-culture with either mock-infected or HIV-infected CD4 T cells (*n* = 20). Markers with adjusted *p* value <0.05 are shown in black and markers with adjusted *p* value >0.05 are shown in gray; adjusted *p* values calculated on GLMM with Benjamini-Hochberg correction to control the false discovery rate. Also see Figure S2.

To identify discrete NK cell receptors that may contribute to the anti-HIV response, we used a generalized linear mixed model (GLMM) implemented through CytoGLMM to compare paired functional^+^ and functional^−^ NK cells in co-culture with HIV-infected cells from each donor (Figure 2A).^38^ For each sample we gated on NK cells that were positive for CD107a, IFN-ɣ, or TNF-ɑ (Figures S2B and S2C), and used Boolean gating to identify functionally responding cells (termed functional^+^, positive for *any* of the functional markers above), or non-functionally responding cells (termed functional^−^, negative for *all* of the markers above). This revealed a distinct phenotype of responding functional cells; with the exception of 5 receptors, every NK cell receptor profiled was a significant predictor of either a functional^+^ or functional^−^ phenotype (Figure 2A). The top three predictors of responding functional^+^ cells were TIGIT, NKp30, and KIR3DL1. Notably, when we conducted the same analysis with NK cells co-cultured with mock-infected CD4 T cells, we found that many of the predictors of responding cells were similar (Figure 2B). This suggests that the responding NK cell phenotype is not unique to infection, likely because the activation of CD4 T cells necessary for infection can upregulate stress ligands and induce NK cell killing.^39^ Indeed, when we compared functional cells from NK cells co-cultured with mock-infected cells to those co-cultured with HIV-infected cells, we found few predictors that distinguished them (Figure 2C). These results suggest that HIV-targeting by NK cells may not rely on distinct receptors but may instead rely on altered levels of signaling from these receptors.

### HIV infection of CD4 T cells induces upregulation of activating NK cell ligands

**Figure 3:**
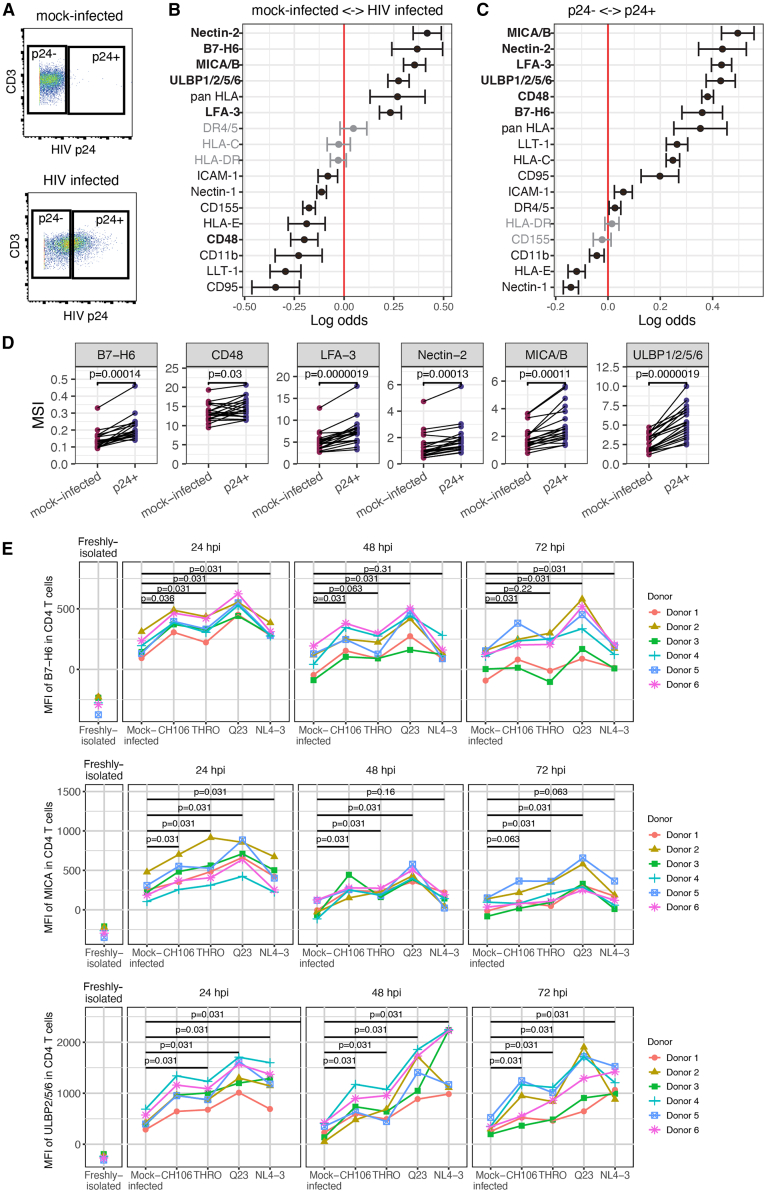
A group of activating NK cell ligands are upregulated in HIV-infected cells Expression of NK cell ligands on CD4 T cells in (A) to (D) was profiled by CyTOF. (A) Representative dot plots from mass cytometry, demonstrating the gating strategy to identify HIV-infected cells as determined by positive staining for HIV p24. (B and C) A generalized linear mixed model (GLMM) with paired comparison was used to compare (B) mock-infected and HIV-infected cells (*n* = 20), and (C) p24^-^ and p24^+^ cells from within infected wells (*n* = 20). Markers with adjusted *p* value <0.05 are shown in black and markers with adjusted *p* value >0.05 are shown in gray; adjusted *p* values calculated on GLMM using Benjamini-Hochberg method to control the false discovery rate. (D) Comparison of mean signal intensity (MSI) between mock-infected and p24^+^ cells for ligands of interest; individual donors are joined by a line. Statistical analysis was performed using the Wilcoxon signed-rank test (*n* = 20). (E) Mean fluorescence intensity (MFI) of B7-H6 (top panel), MICA (middle panel), and ULBP2/5/6 (bottom panel) in flow cytometry analysis of CD4 T cells that are freshly-isolated from PBMCs of healthy donors or at 24, 48, and 72 h post-infection (hpi) with 4 different HIV strains or with mock-infection (*n* = 6). Statistical analysis was performed with the Wilcoxon signed-rank test. Also see Figures S3–S7.

The presence of certain receptors on NK cells responding to HIV infection may merely represent markers of generally more functional NK cells, instead of receptors involved in the specific response to HIV infection. Indeed, the top predictor of responding cells was TIGIT (Figure 2A), which we have reported to mark a more functional NK cell subset.^40^ However, it is still under discussion if TIGIT influences NK cell response that is specific to HIV infection in primary CD4 T cells.^40^^,^^41^^,^^42^^,^^43^ Similarly, KIR3DL1 is known to participate in recognition of HIV-infected cells,^44^ but our goal was to identify receptor:ligand pairs that participate in recognition that would be shared between individuals regardless of genotype. To gain insight into which NK cell ligands on primary CD4 T cells are modulated by HIV infection and thereby trigger HIV-specific NK cell responses, we complemented NK cell receptor profiling with an analysis of NK cell ligand expression on HIV-infected CD4 T cells. We utilized a CyTOF panel that included a set of known activating and inhibitory ligands for NK cell receptors to profile CD4 T cells (gating strategy in Figure S2D) 24 h after the inoculation of Q23 strain (HIV-infected in Figure 3A), as well as mock-infected CD4 T cells (Figure 3A). We also identified actively infected (HIV Gag p24^+^) and bystander (p24^-^) cells in the CD4 T cell culture with HIV (Figure 3A). To understand the alterations in NK cell ligand expression in HIV-infected cells, we used *CytoGLMM* to identify surface ligands that predicted CD4 T cells in the culture with HIV compared to mock-infected cells (Figure 3B). Furthermore, in the CD4 T cell culture inoculated with HIV, we also identified predictors of p24^+^ cells compared to bystander p24^-^ T cells (Figure 3C). We found that Nectin-2 (ligand of DNAM-1), B7-H6 (ligand of NKp30), LFA-3 (ligand of CD2), MICA/B (ligands of NKG2D), and ULBP1/2/5/6 (ligands of NKG2D) were all predictors for infected cells (Figures 3B and 3C); CD48 (ligand of CD244) was a predictor of p24^+^ cells when compared to p24^-^ bystanders (Figure 3C), although not predictive of total CD4 T cells in the culture with HIV compared to mock-infected controls (Figure 3B). The increased expression of these markers was confirmed by analyzing mean signal intensity (MSI), which showed that MSI was higher in p24^+^ cells compared to mock-infected cells for all these ligands (Figure 3D). Notably, these are all activating ligands for NK cells through interaction with their receptors; the expression of the corresponding receptors on NK cells, including NKp30 (receptor for B7-H6) and CD244 (receptor for CD48), was also higher on functionally responding NK cells (Figure 2A) revealing potential pathways for NK cell recognition of HIV-infected cells. Thus, using a combination of CyTOF screens on NK cell receptors and on their ligands expressed by CD4 T cells, we identified a set of 6 candidate receptor-ligand pairs that had a potential role in NK cell recognition of HIV-infected cells: DNAM-1:Nectin-2, NKp30:B7-H6, CD2:LFA-3, CD244:CD48, NKG2D:MICA/B, and NKG2D:ULBP1/2/5/6.

### The pattern of NK cell ligand expression in HIV-infected CD4 T cells varies with different virus strains and among individuals

To further understand whether different strains of HIV might induce variable patterns of NK cell ligand alteration therefore differentially influence NK cell recognition, we compared ligand expression on CD4 T cells infected with each of 6 HIV strains (Figure S3A), using the same ligand panel for CyTOF analysis as aforementioned. These strains included Q23, a commonly used lab-adapted strain NL4-3, as well as a panel of T/F strains, which are particularly relevant for the study of early innate immune cell responses. To look at overall NK cell ligand expression patterns on total infected cells (p24^+^, pre- and post-integration) only, we used a principal-components analysis (PCA) plot to visualize all samples colored by virus strain (Figure S3B top left panel) and blood donor (Figure S3B bottom left panel). We found that samples grouped predominantly based on blood donor (Figure S3B) suggesting a greater impact of blood donor than of virus strain on the overall pattern of NK cell ligand expression. To better understand the differences in NK cell ligand modulation driven by donor variability, compared to driven by different viral strains, we performed unsupervised hierarchical clustering based on mean marker expression for all profiled ligands in p24^+^ cells from each sample (Figure S3C). We again found that samples clustered by donor but not viral strain, reinforcing that patterns of NK cell ligand expression are driven predominantly by donor-to-donor variance. To verify whether ligands upregulated in Q23 infection were shared across other HIV strains, we compared individual ligand expression across all the 6 HIV strains. CD48, LFA-3 and MICA/B were differentially regulated by different strains while the upregulation of B7-H6, ULBP1/2/5/6, and Nectin-2 in the infected CD4 T cells are conserved among Q23 and all 4 T/F strains in most of the donors (Figure S3D). Expression levels of MICA/B and ULBP1/2/5/6 were higher in CD4 T cells infected with Q23 than with all the subtype B T/F strains in most of the donors (Figure S3D). We also observed similar patterns of NK cell ligand upregulation in p24^+^ cells infected with Q23, NL4-3, CH106 2633 (shortened as CH106), and THRO 2626 (shortened as THRO) at later time points, including 48 and 72 h post-infection (hpi) (Figures 3E, S4, and S5). Importantly, although the expression levels of B7-H6, MICA, MICB, and Nectin-2 are lower in Gag p24^+^CD4^−^ (post-integration) cells than Gag p24^+^CD4^+^ (pre-integration) cells for some donors, likely due to modulation by viral accessory proteins post-integration, their levels of expression in Gag p24^+^CD4^−^ CD4 T cells were still higher than bystander CD4 T cells (Gag p24^−^CD4^+^) (Figures S6B, S6E, S7A, and S7D–S7F) at 72 hpi. The expression levels of ULBP1 and ULBP2/5/6 are higher in Gag p24^+^CD4^−^ (post-integration) T cells than Gag p24^+^CD4^+^ (pre-integration) T cells for most of the donors (Figures S6H, S6I, S7G, and S7H). Collectively, our data suggest that NK cell ligand expression can be differentially shaped by different HIV strains yet highly variable among individuals.

### Knockout of B7-H6 and MICB diminish the HIV-specific NK cell response

**Figure 4:**
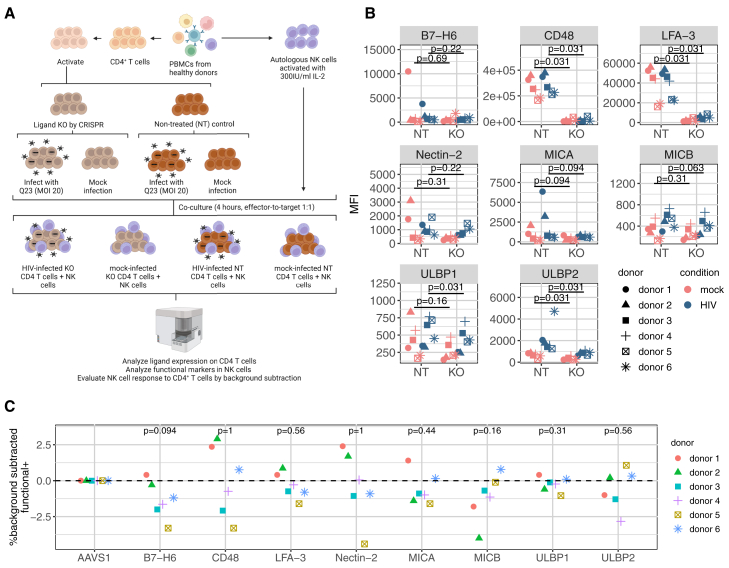
Knockout of NK cell ligands in CD4 T cells reveals that reduction of B7-H6 or MICB reduces the HIV-specific NK cell response (A) A schematic of the experimental design for testing the function of NK cells in response to CD4 T cells in which individual NK cell ligands were knocked out with CRISPR. This image was created with BioRender.com. (B) Mean fluorescence intensity (MFI) of each ligand tested, as well as non-treated (NT) control, in mock- (in pink) and HIV-infected (Gag p24^+^ in blue) CD4 T cells. Each donor is indicated by a different symbol. *n* = 6. Statistical analysis was performed with the Wilcoxon signed-rank test to compare the expression of NK cell ligands in mock (or HIV)-infected non-treated and cells knocked out of each individual ligand. (C) Quantification of the HIV-specific effect of knocking out each ligand within each donor. For each ligand knockout, we determined the HIV-specific response by subtracting the percentage of functional^+^ NK cells from the mock-infected CD4 T cells from the percentage of functional^+^ NK cells from the HIV-infected CD4 T cells. We then normalized the HIV-specific response for each donor to the control AAVS1-edited group by subtracting that level, such that negative values indicate a diminished functional response and positive values indicate an enhanced functional response compared to the control AAVS1-edited group. Each donor is indicated by a different symbol and color. *n* = 6. Statistical analysis was performed with the Wilcoxon signed-rank test to compare each knockout group with the AAVS1-edited group. Also see Figures S8 and S9.

To assess the individual roles of each of these ligands, we knocked each of them out separately in CD4 T cells, and then probed the NK cell response in the presence or absence of HIV infection (Figure 4A). CD4 T cells were electroporated with CRISPR-Cas9 ribonucleoproteins (RNPs) containing guide RNAs (gRNAs) against each ligand of interest. We also included non-treated controls (NT), which were not electroporated, as well as a control targeting AAVS1, a “safe harbor” region of the genome, which can be edited without causing discernible negative impact on the cells.^45^ Targeting of AAVS1 did not lead to any changes in ligand expression compared to NT controls, indicating that RNP electroporation or targeted double-strand breaks in the genome did not lead to any modulation of ligand expression (Figures S8A and S8B). To test for the efficiency of ligand knockout, we examined surface expression of each ligand 8 days post-electroporation by flow cytometry, in both mock- and HIV-infected cells. Despite considerable variability between donors in the level of NK cell ligand expression both before and after knockout, we noted an expected reduction in the expression level of most ligands in targeted knockouts compared to NT in HIV-infected and mock-infected CD4 T cells (Figures 4B, S8C, and S8D) in most of the donors. The reduction in B7-H6, Nectin-2, MICA, MICB, ULBP1, and ULBP2 (evaluated with an antibody that binds to ULBP2 and cross-reacts with ULBP5 and 6) was mild in some or all the donors compared to CD48 and LFA-3, possibly due to their low level of expression even in non-treated CD4 T cells (Figures 4B, S8C, and S8D). CRISPR editing did not lead to a discernible effect on T cell susceptibility to HIV infection (Figure S9A).

To identify the effects of these ligand knockouts on NK cell responses in the context of HIV infection, we co-cultured edited CD4 T cells that were either mock-infected or Q23-infected with autologous NK cells, and then monitored the NK cell functional response using flow cytometry (Figure 4A). We used the frequency of functional NK cells as defined above (% functional^+^) to evaluate overall NK cell responses. Unexpectedly, in some donors, knockout of CD48, MICA, and MICB, led to increased functional responses compared to NT controls in response to both mock- and HIV-infected cells (Figure S9B). As we were predominantly interested in HIV-specific responses, we applied mock-subtraction to identify HIV-specific responses, and donor-level normalization to evaluate the effect of knocking out each NK cell ligand within each donor by comparing them to the control group where AAVS1 was edited (Figure 4C). Despite the low level of expression in non-treated CD4 T cells, knockout of B7-H6 (NKp30 ligand) and MICB (NKG2D ligand) resulted in a trend toward less HIV-specific NK cell responses, which were variable across the donors (Figure 4C). However, knockout of any of the ligands did not lead to a complete abrogation of HIV-specific NK cell targeting except for one donor (donor 5) when B7-H6 or CD48 or Nectin-2 was knocked out (Figure S9B). As such, while knockout of B7-H6 and MICB diminished the NK cell response toward HIV-infected CD4 T cells from some donors, knockout of any individual NK cell ligand did not fully abolish the HIV-specific response.

### Overexpression of NKp30 and NKG2D enhances NK cell response to HIV-infected CD4 T cells

Since all HIV strains tested led to upregulation of ULBP1/2/5/6 and B7-H6 on HIV-infected CD4 T cells, and the knockout of B7-H6 and MICB modestly reduced NK cell responses to infected cells in most donors, we further investigated if their receptors, NKp30 and NKG2D, influence NK cell response. We also investigated the role of CD244 (2B4) in NK cell response to CD4 T cells since CD244 is among the top predictors of functional NK cells in the co-culture with both Q23-infected and mock-infected CD4 T cells (Figures 2A and 2B) yet knockout of its ligand, CD48, led to variable effects on the HIV-specific NK cell response in different donors (Figure 4C). We overexpressed NKp30, NKG2D, and CD244 in NK cells with charge-altering releasable transporter (CART) transfection. CART transfection allows highly efficient delivery and expression of exogenous mRNA in human primary NK cells, preserving their viability and causing minimal impact on their phenotypes.^46^^,^^47^ We isolated NK cells from PBMCs of healthy donors and transfected them with CART and the encoding mRNAs of NKp30, NKG2D, and CD244. To distinguish the NK cells that overexpress these receptors after transfection and the ones that were not transfected but naturally express the receptors, we co-transfected the NK cells with GFP mRNA to indicate the translation of exogenous mRNAs. Since DAP10 is required for the surface expression of human NKG2D and serves as a signaling adaptor of NKG2D,^48^^,^^49^ we first ascertained if excessive DAP10 mRNA was required for surface expression of exogenous NKG2D in human NK cells. Interestingly, the endogenous level of DAP10 in NK cells is not enough to support the surface expression of the exogenous NKG2D (Figures S10A–S10C). Co-transfection of NKG2D and DAP10 mRNAs at a ratio of 3:1 maximizes the expression level of NKG2D in NKG2D^+^GFP^+^ double-positive NK cells (Figure S10C).

**Figure 5:**
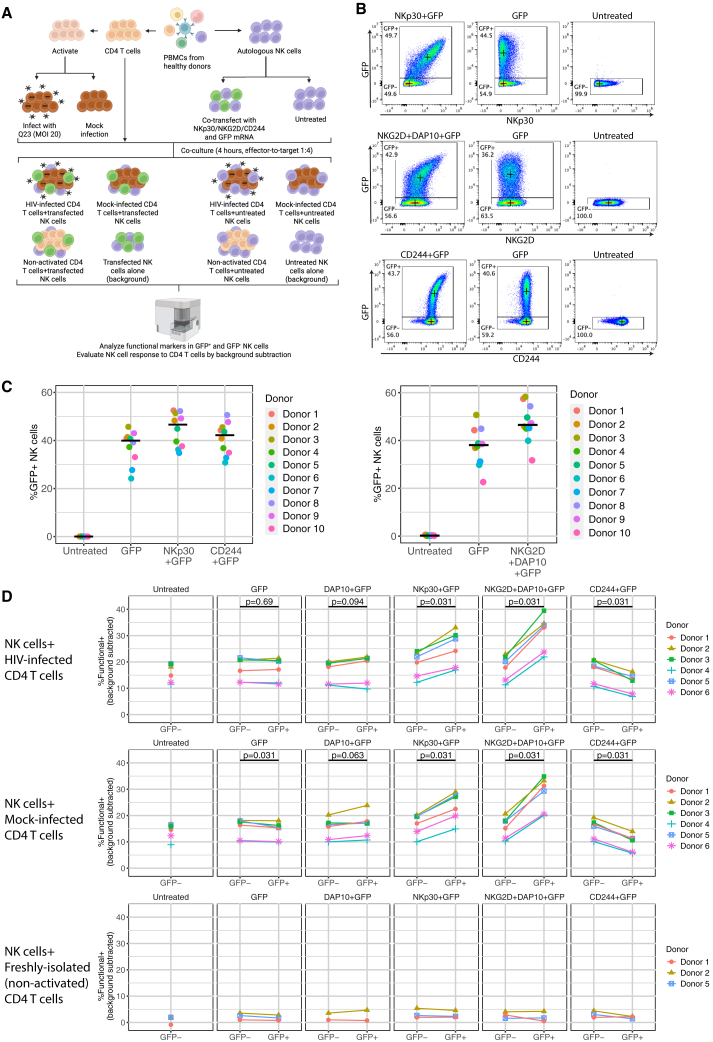
Overexpression of NKp30 or NKG2D enhances NK cell functional response to HIV-infected CD4 T cells (A) Schematic depicting the experimental design of testing the function of transfected NK cells in response to autologous HIV-infected CD4 T cells and mock-infected CD4 T cells. This image was created with BioRender.com. (B) Representative flow cytometry plots indicating the co-expression of GFP and each of the co-transfected NK cell receptors (NKp30, NKG2D, and CD244). Crosses indicate the median fluorescence intensity of the marker on x and y axes in each gate. (C) Quantification of the percentage of GFP^+^ NK cells as in (B). *n* = 10. Bars indicate the median of each group. (D) Percentage of functional^+^ NK cells (positive for any of CD107a, IFN-ɣ, and TNF-ɑ) in GFP^+^ and GFP^−^ populations after transfection with the mRNA as labeled and co-cultured with autologous Q23-infected (top panel), mock-infected (middle panel), and non-activated CD4 T cells (bottom panel) after subtracting the percentage of functional^+^ NK cells in NK alone group (background subtraction). n = 2–6. Statistical analysis was performed with the Wilcoxon signed-rank test. Also see Figures S10 and S11.

We evaluated if NK cell function in response to Q23-infected CD4 T cells is altered with the overexpression of NKp30, NKG2D, or CD244 (Figure 5A). At 16–19 h after NK cells were transfected with each of the three combinations of encoding mRNA, including NKp30+GFP, NKG2D+DAP10+GFP, and CD244+GFP, we observed a population of GFP^+^ NK cells (30%–60%, Figures 5B and 5C) that expressed higher levels of NKp30, NKG2D, or CD244, respectively, compared to the GFP^−^ NK cells in the same well (Figure 5B). We co-cultured transfected NK cells (including both GFP^−^ and GFP^+^ in the same well) in each of the three groups mentioned above with autologous Q23-infected or mock-infected CD4 T cells at E:T ratio of 1:4 for 4 h. NK cells that were cultured alone serve as controls that indicate the background level of NK cell function. When NK cells were co-transfected with combinations of mRNA that encode either NKp30+GFP or NKG2D+DAP10+GFP, the percentage of functional^+^ NK cells (as defined above, gating strategy in Figure S10D) in the GFP^+^ population was significantly higher than in the GFP^−^ population in the same well in the presence of Q23-infected CD4 T cells (Figure 5D top panel). When NKs were co-transfected with CD244 and GFP, the percentage of functional^+^ in the GFP^+^ population was significantly reduced compared to the GFP^−^ population (Figure 5D top panel). The alteration of function in GFP^+^ NK cells is not due to the expression of GFP or excessive amount of DAP10 because transfection of GFP mRNA alone or co-transfection with DAP10 and GFP only marginally influenced the percentage of functional^+^ NK cells (Figure 5D top panel). Furthermore, when we stratified NK cells based on the expression level of NKp30, NKG2D, or CD244 to high, intermediate and low/negative (Figure S11A), we noticed positive correlations between the percentage of functional^+^ NK cells and the expression level of NKp30 or NKG2D (Figures S11B and S11C, top panels). We also observed that the percentage of functional^+^ NK cells in the CD244^high^ population was reduced compared to the CD244^int^ and CD244^low/-^ populations (Figure S11D, top panel). However, the alteration of NK cell function due to NKp30, NKG2D, and CD244 overexpression is not specific to Q23 infection, as we also observed similar responses to mock-infected targets. When co-cultured with mock-infected CD4 T cells, we observed an elevated percentage of functional^+^ NK cells when they overexpress NKp30 or NKG2D (Figure 5D middle panel, S11B and C, bottom panels) and reduced percentage of functional^+^ NK cells when they overexpress CD244 (Figures 5D middle panel, S11D bottom panel). Notably, only 0%–5% of NK cells were functional^+^ in both GFP^+^ and GFP^−^ populations when co-cultured with resting CD4 T cells (freshly isolated from PBMCs without stimulation) (Figure 5D bottom panel), indicating that this enhanced activity required B7-H6, MICA/B, or ULBP1/2/5/6 expression, which occurs on activated, but not resting, CD4 T cells.

### Blockade of NKp30 and NKG2D compromises the specificity of NK cell cytotoxicity on HIV-infected CD4 T cells

**Figure 6:**
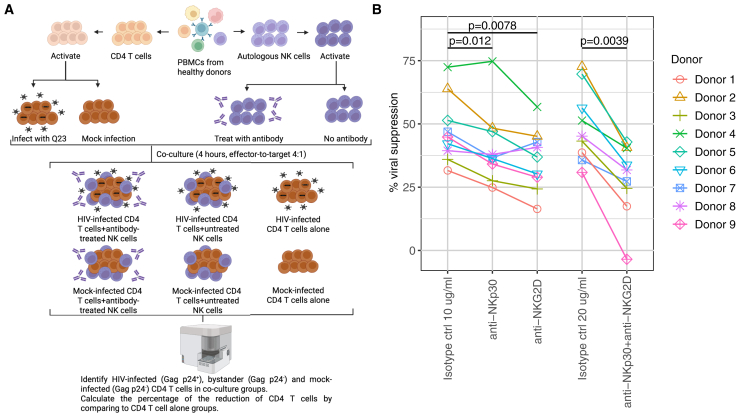
Blockade of NKp30 and NKG2D in NK cells reduces HIV-specific cytotoxicity on CD4 T cells (A) Schematic depicting the experimental design of testing the cytotoxicity of NK cells treated with anti-NKp30 or anti-NKG2D blocking antibodies on autologous HIV-infected CD4 T cells and mock-infected CD4 T cells. This image was created with BioRender.com. (B) Percentage of the reduction of the percentage of HIV-infected CD4 T cells (Gag^+^) after co-cultured with NK cells compared to CD4 T cells that were cultured alone. The value of each data point is an average of 2–3 technical replicates. *n* = 9. Statistical analysis was performed with the Wilcoxon signed-rank test. Also see Figure S12.

Given the increase of functional markers in NK cells that overexpress NKp30 or NKG2D when co-cultured with CD4 T cells, we directly evaluated the role of these receptors in the cytotoxicity of NK cells on HIV-infected and bystander CD4 T cells. Since serum-free treatment, which is necessary for CART transfection, causes reduction of granzyme B expression (data not shown) and potentially compromises the killing capacity of NK cells, we investigated the function of NKp30 and NKG2D in HIV-specific NK cell cytotoxicity by blocking the receptors with antibodies instead. We treated IL-2-stimulated NK cells with anti-NKp30 and anti-NKG2D antibodies individually or in combination for 2 h, and co-cultured them with autologous Q23-infected or mock-infected CellTrace Violet-labeled CD4 T cells at E:T ratio of 4:1 for 4 h (Figure 6A). NK cells that were treated with the isotype control of both antibodies at the same concentration served as controls. Viable Q23-infected cells (Gag p24^+^) and bystander cells (Gag p24^-^) as well as Gag p24^-^ mock-infected CD4 T cells were identified with flow cytometry (Figures S12A and S12B). Compared to Q23-infected CD4 T cells that were cultured alone (in the absence of NK cells), co-culture with NK cells caused reduction in the percentage of Gag p24^+^ cells due to the preferential elimination of Gag p24^+^ cells mediated by NK cell cytotoxicity (Figures 1D and 6B). The reduction of the percentage of Gag p24^+^ cells decreased significantly when NK cells were treated with anti-NKp30 and anti-NKG2D antibodies either individually or in combination (Figures 6B and S12C), indicating that blockade of these two receptors compromised the specificity of HIV targeting. Consistently, the reduction of the Gag p24^+^ CD4 T cell count in the co-culture with NK cells treated with anti-NKp30 antibody, anti-NKG2D antibody or a combination decreased in a majority of donors compared to NK cells treated with the isotype control (Figure S12D). Importantly, killing of Gag p24^-^ bystander or mock-infected CD4 T cells was not altered significantly in the co-culture groups where NK cells were treated with anti-NKp30 or anti-NKG2D antibodies compared to isotype control (Figure S12D). These data suggest that NKp30 and NKG2D facilitate the specificity of NK cell cytotoxicity on Gag p24^+^ CD4 T cells. Together, these data suggest that NKp30:B7-H6 and NKG2D:MICB receptor-ligand pairs facilitate NK cell recognition of HIV.

## Discussion

In this study, we investigated the mechanism of NK cell recognition of autologous CD4 T cells at the early stage after HIV-1 infection. We use mass cytometry to profile in-depth the NK cell response to autologous HIV-infected cells; this encompasses both the phenotypic features of responding NK cells as well as the modulation of cognate NK cell ligand expression on infected CD4 T target cells. To identify the NK cell receptors most important for the response to HIV-infected cells, we further used mass cytometry to characterize both NK cell receptor and ligand expression during NK-CD4 T cell co-cultures. We find that HIV infection induces upregulation of a number of surface proteins that are ligands for activating NK cell receptors, including Nectin-2, B7-H6 and the various ligands of the NKG2D receptor, though there is considerable variation between donors in the levels of these ligands. Of these, we observed reduced functional NK cell responses to infected cells when eliminating the interactions between NKp30 and its ligand B7-H6, and eliminating the interaction between NKG2D and its ligand MICB in some donors. Importantly, no single knockout of the ligands or antibody blockade of NKp30 or NKG2D was able to fully abrogate the NK cell response to HIV-infected cells, suggesting redundancy in recognition mechanisms. Consistent with this observation, increasing the interaction between NKp30 and NKG2D and their ligands by overexpressing NKp30 or NKG2D increased NK cell functional response on HIV-infected cells.

We first optimized an *in vitro* co-culture system to detect NK cell response to HIV-infected cells. It has been previously reported that, *in vitro*, HIV-infected CD4 T cells escape NK cell killing via a number of viral escape pathways mediated by accessory proteins.^15^^,^^50^ However, *in vivo*, epidemiological evidence suggests that NK cells can indeed contribute to viral clearance and mediate protection from infection and disease progression, suggesting that *in vitro* methods may require optimization for the effective detection of HIV-specific responses. The use of IL-2 to pre-activate NK cells is known to enhance their ability to lyse infected cells^51^; as such, we used IL-2 activated NK cells to maximize the ability of NK cells to target infected cells, thereby allowing us to probe the specificities of these interactions. While we detected a response to activated, mock-infected autologous CD4 T cells, we also consistently observed a greater response to HIV-infected cells, suggesting that HIV-1 infection drove additional ligand modulation pathways that allowed further targeting by NK cells. Using mass cytometry to compare ligand expression between infected cells, bystander cells and mock-infected cells, we were able to further identify candidate ligands that contributed to recognition of HIV-1 by NK cells, and among these, we demonstrate that overall patterns of B7-H6 and ULBP1/2/5/6 expression were similar across multiple strains of HIV of different subtypes, though there was considerable variation between donors in their expression levels.

We did not account for the effects of KIR and HLA genotypes in our assay, as our primary focus was on identifying shared pathways that were independent of donor genotypes, and hence more broadly applicable in the development of vaccines and therapeutics. Numerous groups have reported the contribution of variation in KIRs and HLAs to both NK cell education and resultant functionality,^18^ as well as HIV-targeting NK cell activity. As such, different KIR and HLA haplotypes may contribute to the high degree of variation in NK cell responses observed between donors (Figure 1). However, in our CyTOF screens for receptors that mark HIV-responsive NK cells, the only KIR that was a predictor of functional NK cells was KIR3DL1 (Figure 2A), and it was not a predictor of HIV specificity (vs. mock-infected) (Figure 2C). This is consistent with previous reports that KIR3DL1^+^ cells are more functional but are not capable of targeting HIV.^12^^,^^52^

Lucar et al. reported upregulation of the NKp30 ligand B7-H6 for HIV-2; the authors also showed an upregulation of a NKp30 ligand in HIV-1 infected cells, though to a lower extent.^27^ We observed a small upregulation of B7-H6 in p24^+^ cells compared to mock-infected (Figure 3D), which was supported by the fact that NKp30 was also a predictor of cells responding to HIV-infected cells. In addition, knocking out B7-H6 by CRISPR partially abrogated HIV-specific NK cell response in some donors (Figure S9B). Overexpression of NKp30 in NK cells elevated NK cell response to Q23-infected CD4 T cells (Figure 5D) and blockade of NKp30 compromised the specificity of NK cell response to Q23-infected CD4 T cells (Figure 6B). Although blockade of NKp30 with antibodies had only marginal and donor-to-donor variable impact on the elimination of Gag p24^+^ cells compared to the blockade of NKG2D (Figure S12D) possibly due to the low expression level of the ligand of NKp30, blockade of NKp30 caused a significant increase in the percentage of Gag p24^+^ cells after co-culture compared to isotype control treatment (Figure S12C), indicating that NKp30 facilitates the specificity of NK cell targeting on the infected cells. These data suggest that B7-H6 can indeed contribute to recognition of HIV-1, similar to HIV-2. However, our ligand panel is limited to the detection of a subset of known ligands for each receptor, and it is unclear if additional NKp30 ligands besides B7-H6, such as BAT3 and galectin-3,^53^ may also contribute to recognition of HIV-1. This will be an important area of further investigation.

Among the various NKG2D ligands, ULBPs have been previously reported to be upregulated in HIV-1-infected cells and can trigger NK cell-mediated lysis.^23^^,^^54^^,^^55^ MICA and MICB have also been reported to be upregulated in primary CD4 T cells that are infected with HIV *in vitro*, or in a latently-infected cell line that have been reactivated with latency-reversal agent.^56^^,^^57^ Soluble NKG2D ligands shed from the infected cells can cause downregulation of NKG2D in NK cells.^57^ Other groups have reported marginal or no upregulation of MICA or MICB in CD4 T cells that are transduced with HIV-1 vpr or isolated from HIV patients.^23^^,^^51^^,^^54^ Here, we confirm that expression of ULBP1/2/5/6 is higher in p24^+^ cells than p24^-^; additionally, we find that the expression of MICA/B is also increased (Figures 3C–3E, S4B, S4C, S5D, and S5E). The upregulation of MICA/B is possibly specific to certain HIV strains or subtypes since infection with different strains led to different levels of upregulation of MICA/B in primary CD4 T cells, although we did not evaluate the level of soluble NKG2D ligands in the supernatant of the culture. Our result showing antibody blockade of NKG2D reduces NK cell targeting of HIV-infected cells but does not completely abrogate it is consistent with previous work by other groups (Figures 6B, S12C, and S12D)^23^^,^^54^^,^^55^; similarly, we find that knockout of NKG2D ligand, MICB, in CD4 T cells from most of the donors that were tested induces an incomplete reduction of HIV-specific NK cell responses (Figure S9B) suggesting redundancy, and that all of these ligands contribute to HIV infection recognition by NK cells.

NKp30 and NKG2D overexpression by mRNA and CART transfection enhanced the functional response of NK cells to HIV-infected as well as mock-infected CD4 T cells. Ligands of NKp30 and NKG2D are naturally expressed in tumor, virus-infected and stressed cells, but rarely expressed in normal cells. *In vitro* activation of CD4 T cells can induce the expression of various NKG2D ligands and render CD4 T cells susceptible to NKG2D-mediated NK cell killing.^58^ Here, we activated CD4 T cells with plate-bound α-human CD3 antibodies, PHA-L, and α-human CD28/CD49d antibodies, which render primary CD4 T cells susceptible to HIV infection *in vitro*. Activated yet uninfected (mock-infected) CD4 T cells also express B7-H6, MICA/B, and ULBP1/2/5/6, although to a lower level than p24^+^ cells (Figures 3D, 3E, S4B, S4C, S5D, and S5E), and interact with NKp30 and NKG2D, which possibly explains the elevation of the function of NKp30 and NKG2D-overexpressing NK cells when co-cultured with mock-infected CD4 T cells. Indeed, when we co-cultured the NK cells that overexpress NKp30 or NKG2D with freshly-isolated non-activated CD4 T cells *in vitro*, NK cell function only marginally increased compared to NK cell alone group, probably because natural CD4 T cells in PBMCs of healthy donors do not express the ligands. Thus, the enhanced functional responses to mock-infected cells are likely an artifact of the activation necessary to encourage HIV infection. Nevertheless, NKp30 and NKG2D blockade in NK cells caused elevation in the percentage of Gag p24^+^ T cells compared to when treated with isotype control (Figure S12C), suggesting that NKp30 and NKG2D facilitate the specific targeting of HIV-infected cells.

The broad genetic variability across HIV strains hinders the development of effective adaptive immune responses.^59^^,^^60^ As an innate cell type, NK cells are classically thought to recognize conserved features of pathogens; however, we have previously shown that two strains of influenza can be differentially recognized by NK cells due to differences in ligand modulation.^30^ HIV proteins, many of which are responsible for the modulation of NK cell ligands, have considerable diversity at the amino acid level,^61^ which may lead to differences in ligand modulation between strains. Differences in individual ligands have been reported; for example, the commonly used lab-adapted strain NL4-3 does not downregulate HLA-C expression, contrary to other clinical isolate strains.^14^ Indeed, we also observed NL4-3-specific patterns in expression of NK cell ligands—for example, CD48 expression was distinctly higher on NL4-3-infected cells compared to those infected with other viral strains in the same donor (Figure S3D). However, using PCA as well as unsupervised clustering, we found that overall patterns of NK cell ligand expression were determined more by donor (implicating host genetics) than by infecting strain (Figures S3B and S3C). For instance, upregulation of B7-H6 and ligands of NKG2D (ULBP1/2/5/6) in infected CD4 T cells is conserved among Q23 and all 4 T/F strains (Figure S3D), suggesting this recognition mechanism is shared across multiple HIV strains. Overall, methods to target HIV via NK cell receptors are likely to be broadly applicable across HIV strains of different subtypes; however, lab-adapted HIV strains may not entirely recapitulate relevant features in studies of NK cell targeting.

In conclusion, we have developed an experimental and analytical system to probe receptor-ligand interactions involved in the recognition of autologous early HIV-infected CD4 T cells by NK cells. By simultaneously profiling NK cell receptor expression and functional activity, and combined with NK cell ligand expression on infected cells, we were able to develop a list of putative targets that were then investigated by CRISPR knockout of the ligands and overexpression of some of the receptors. This confirmed prior reports of the roles of NKG2D-MICA/B and ULBP1/2/5/6 interactions, and we newly identified NKp30 interaction with B7-H6 as an important mechanism of recognition of HIV-1-infected T cells. While further work is required to delineate the full set of ligands that are required for recognition, our studies set the stage for rationally modulating NK cell ligands and receptors to improve HIV-targeting activity.

### Limitations of the study

One limitation is that receptors on the surface of NK cells, including NKG2D,^62^^,^^63^^,^^64^ NKp30,^65^ and DNAM-1 can be downregulated after persistent binding to their ligands.^66^^,^^67^ As such, profiling cannot distinguish receptors that are lowly or not expressed on functional cells from those that are used but downregulated after ligand engagement. This is why we relied on knockout of individual NK cell ligands (with caveats therein), over-expression of NKp30 and NKG2D and antibody blockade of the receptors to interrogate the roles of these receptor-ligand interactions. Another limitation of our study is that we were not able to confirm that knockout of any one NK cell ligand does not impact the expression of others during HIV infection. As such, further studies profiling the entire NK cell ligand repertoire are required in these edited T cells to better understand potential compensatory mechanisms that may contribute to the limited effects of ligand knockout on NK cell responses we observed (Figure 4C). In addition, knockout of multiple ligands at the same time could delineate the effects of receptor-ligand interactions whose pathways are shared or redundant. In particular, MICA, MICB, ULBP1, and ULBP2 can all induce signaling through the NKG2D receptor, and this redundancy may dampen the effects of knockout of any single ligand.^68^ In addition, our screening of NK cell ligands on infected cells did not account for recognition mechanisms based on potential changes in the peptide repertoire presented on HLA class I molecules in the context of infection. Recent work in non-human primates, as well as humans, have identified SIV/HIV infection-induced alterations in peptides presented on MHC class I,^69^^,^^70^ suggesting that this may also be an important determinant of NK cell triggering.

The phenotype of NK cells is altered by HIV-1 infection and anti-retroviral therapy (ART).^40^^,^^71^ In this work, we investigated the mechanism of HIV recognition with NK cells and CD4 T cells from the peripheral blood of healthy donors. Further studies that profile the NK cells and HIV-infected cells in people living with HIV and in tissues are required to provide more confirmatory evidence for the mechanism of HIV recognition in patients.

## Resource availability

### Lead contact

Further information and requests for resources and reagents should be directed to and will be fulfilled by the lead contact, Dr. Catherine A. Blish (cblish@stanford.edu).

### Materials availability

This study did not generate new unique reagents.

### Data and code availability

• FCS files of all the CyTOF and flow cytometry analysis in this study have been deposited at FlowRepository or Cytobank Community and are publicly available as of the date of publication.^72^
• This paper does not report original code.
• Any additional information required to reanalyze the data reported in this paper is available from the lead contact upon request.

## Acknowledgments

This work was supported by the 10.13039/100000002National Institutes of Health, the United States (NIH DP1 DA046089 and NIH R01 AI161803, to C.A.B., and NIH K08 AI153767 to D.N.N.), a pilot project through the 10.13039/100000865Bill & Melinda Gates Foundation, the United States (OPP1113682, C.A.B.), and the National Science Scholarship from A∗STAR Singapore (N.Q.Z.). The Marson lab support includes funding from 10.13039/100000002NIH the HIV Accessory & Regulatory Complexes (HARC) Center, the United States (P50 AI150476), 10.13039/100000002NIH/ 10.13039/100000060NIAID, the United States P01AI138962, Gilead, and The 10.13039/100000893Simons Foundation, the United States. We thank Drs. Paul A. Wender, Jiuzhi Sun and Harrison P. Rahn in Stanford University for providing the CART ONA used in this work. We thank Dr. Matthew D. Marsden in UC Irvine for advice on the protocol of raltegravir treatment on CD4 T cells. In addition, we thank the Human Immune Monitoring Core (HIMC) at Stanford University for the use of their Helios mass cytometer.

## Author contributions

Conceptualization, R.P., N.Q.Z., and C.A.B.; methodology, R.P., N.Q.Z., and D.N.N.; investigation, R.P., N.Q.Z, A.J.B., T.R., and D.N.N.; formal analysis, R.P., N.Q.Z., C.S., and S.H.; software, C.S., and S.H.; visualization, R.P., and N.Q.Z.; writing – original draft, R.P., and N.Q.Z.; writing – review and editing, C.S., S.H., A.M., D.N.N., and C.A.B.; funding acquisition, A.M., D.N.N., and C.A.B.; resources, A.M. and D.N.N.; project administration, T.R.; supervision, S.H., A.M., and C.A.B.

## Declaration of interests

C.A.B. is a scientific advisory board member of ImmuneBridge, DeepCell, Inc., and Qihan Bio on topics unrelated to this manuscript. A.M. is a cofounder of Site Tx, Arsenal Biosciences, Spotlight Therapeutics and Survey Genomics, serves on the boards of directors at Site Tx, Spotlight Therapeutics and Survey Genomics, is a member of the scientific advisory boards of network.bio, Site Tx, Arsenal Biosciences, Cellanome, Spotlight Therapeutics, Survey Genomics, NewLimit, Amgen, and Tenaya, owns stock in network.bio, Arsenal Biosciences, Site Tx, Cellanome, Spotlight Therapeutics, NewLimit, Survey Genomics, Tenaya and Lightcast and has received fees from network.bio, Site Tx, Arsenal Biosciences, Cellanome, Spotlight Therapeutics, NewLimit, Abbvie, Gilead, Pfizer, 23andMe, PACT Pharma, Juno Therapeutics, Tenaya, Lightcast, Trizell, Vertex, Merck, Amgen, Genentech, GLG, ClearView Healthcare, AlphaSights, Rupert Case Management, Bernstein and ALDA. A.M. is an investor in and informal advisor to Offline Ventures and a client of EPIQ. The Marson laboratory has received research support from the Parker Institute for Cancer Immunotherapy, the Emerson Collective, Arc Institute, Juno Therapeutics, Epinomics, Sanofi, GlaxoSmithKline, Gilead and Anthem and reagents from Genscript, Illumina, and Cellanome.

## STAR★Methods

### Key resources table

**Table:** 

REAGENT or RESOURCE	SOURCE	IDENTIFIER
**Antibodies**		

Purified anti-human CD57 (HCD57)	BioLegend	Cat# 322302; RRID: AB_535988
Qdot-CD19 (SJ25-C1)	ThermoFisher Scientific	Cat# Q10179; RRID: AB_10375451
Purified anti-human CD3 (UCHT1)	BioLegend	Cat# 300402; RRID: AB_314056
Granzyme B monoclonal antibody (GB11)	ThermoFisher Scientific/Invitrogen	Cat# MA1-80734; RRID: AB_931084
Mouse anti-human MIP-1β (D21-1352)	BD Biosciences	Customized order
Human NKG2C/CD159c antibody (134522)	R&D Systems	Cat# MAB1381-100; RRID: AB_2132981
Purified mouse anti-human CD161 (KLRB1) (DX12)	BD Biosciences	Cat# 556079; RRID: AB_396346
Purified anti-human CD38 (HIT2)	BioLegend	Cat# 303502; RRID: AB_314354
Purified anti-human CD8 (SK1)	BioLegend	Cat# 344702; RRID: AB_1877104
Purified anti-allophycocyanin (APC) Maxpar® ready antibody (APC003), for detecting APC-CD107a	BioLegend	Cat# 408005; RRID: AB_2563706
Purified anti-human LFA-1 (CD11a/CD18) (M24)	BioLegend	Cat# 363402; RRID: AB_2564213
Purified anti-human CD2 (LFA-2) (RPA-2.10)	BioLegend	Cat# 300202; RRID: AB_314026
Anti-HIV p24 antigen (39/5.4A)	Abcam	Cat# ab9071; RRID: AB_306981
Purified anti-human CD328 (Siglec-7) (S7.7)	BioLegend	Cat# 347702; RRID: AB_2189411
Anti-Perforin antibody (B-D48)	Abcam	Cat# ab47225; RRID: AB_2169084
Human KIR2DS4 (CD158i) antibody (179315)	R&D Systems	Cat# MAB1847; RRID: AB_2130820
Human LILRB1 (ILT-2/CD85j) antibody (292319)	R&D Systems	Cat# MAB20172; RRID: AB_2249954
Purified anti-human NKp46 (CD335) (9E2)	BioLegend	Cat# 331902; RRID: AB_1027637
Purified anti-human NKG2D (1D11)	BioLegend	Cat# 320802; RRID: AB_492956
Human TIGIT antibody (741182)	R&D Systems	Cat# MAB7898; RRID: AB_3331639
Purified anti-human CD244 (2B4) (C1.7)	BioLegend	Cat# 329502; RRID: AB_1279194
Purified mouse anti-human CD226 (DNAM-1) (DX11)	BD Biosciences	Cat# 559787; RRID: AB_397328
Purified mouse anti-human IFN-γ (B27)	BD Biosciences	Cat# 554699; RRID: AB_398579
Purified anti-human NKp30 (CD337) (P30-15)	BioLegend	Cat# 325202; RRID: AB_756106
TNF-ɑ monoclonal antibody (MAB11)	ThermoFisher Scientific/eBioscience	Cat# 14-7349-85; RRID: AB_468490
Purified mouse anti-human KIR3DL1 (NKB1) (DX9)	BD Biosciences/BD Pharmingen	Cat# 555964; RRID: AB_396258
Purified anti-human NKp44 (CD336) (P44-8)	BioLegend	Cat# 325102; RRID: AB_756094
Purified anti-human CD96 (TACTILE) (NK92.39)	BioLegend	Cat# 338402; RRID: AB_1279386
Human KIR2DL1/KIR2DS5 antibody (143211)	R&D Systems	Cat# MAB1844; RRID: AB_2130400
Purified anti-human CXCL10 (IP-10) (J034D6)	BioLegend	Cat# 519503; RRID: AB_2561408
Purified anti-human CD62L (DREG-56)	BioLegend	Cat# 304802; RRID: AB_314462
Anti-human CD159a/NKG2A (Z199)-169Tm	Standard Biotools	Cat# 3169013B; RRID: AB_2756426
Rabbit anti-human KIR2DS2 antibody (Polyclonal)	Abcam	Cat# Ab175486; currently not available
Purified anti-human PD1 (CD279) (EH12.2H7)	BioLegend	Cat# 329902; RRID: AB_940488
Purified anti-human CD352 (NTB-A) (NT-7)	BioLegend	Cat# 317202; RRID: AB_571931
Purified mouse anti-human CD56 (NCAM16.2)	BD Biosciences/BD Pharmingen	Cat# 559043; RRID: AB_397180
Human KIR2DL3/CD158b2 Antibody (180701)	R&D Systems	Cat# MAB2014; RRID: AB_2130575
Purified anti-human CD69 (FN50)	BioLegend	Cat# 310902; RRID: AB_314837
Anti-human CD16 (3G8)-209Bi	Standard Biotools	Cat# 3209002B; RRID: AB_2756431
Purified anti-human HLA-DR (L243)	BioLegend	Cat# 307602; RRID: AB_314680
Purified anti-human HLA-A,B,C (pan-HLA class I) (W6/32)	BioLegend	Cat# 311402; RRID: AB_314871
Purified anti-human CD7 (CD7-6B7)	BioLegend	Cat# 343102; RRID: AB_1659214
Purified anti-human CD48 (BJ40)	BioLegend	Cat# 336702; RRID: AB_1227561
Purified anti-human CD54 (ICAM1) (HA58)	BioLegend	Cat# 353102; RRID: AB_11204426
Human OCIL/CLEC2d (LLT-1) antibody (402659)	R&D Systems	Cat# AF3480; RRID: AB_2292099
Purified anti-human CD4 (OKT4)	BioLegend	Cat# 317402; RRID: AB_571963
Anti-HLA-C antibody (DT9)	Millipore	Cat# MABF233; RRID: AB_2687888
Purified anti-human CD192 (CCR2) (K036C2)	BioLegend	Cat# 357202; RRID: AB_2561851
Purified anti-human HLA-E (3D12)	BioLegend	Cat# 342602; RRID: AB_1659247
Purified anti-human CD95 (Fas) (DX2)	BioLegend	Cat# 305602; RRID: AB_314540
Purified anti-human Nectin-1 (CD111) (R1.302)	BioLegend	Cat# 340402; RRID: AB_2284683
Human MICA antibody (159227)	R&D Systems	Cat# MAB1300-100; RRID: AB_2143622
Human MICB antibody (236511)	R&D Systems	Cat# MAB1599-100; RRID: AB_2143772
Purified anti-human DR4 (CD261, TRAIL-R1) (DJR1)	BioLegend	Cat# 307202; RRID: AB_314666
Purified anti-human DR5 (CD262, TRAIL-R2) (DJR2-2(2-6))	BioLegend	Cat# 307302; RRID: AB_314672
Human ULBP1 antibody (170818)	R&D Systems	Cat# MAB1380-100; RRID: AB_2214683
Human ULBP2/5/6 antibody (165903)	R&D Systems	Cat# MAB1298-100; RRID: AB_2214692
Purified anti-human Nectin-2 (CD112) (TX31)	BioLegend	Cat# 337402; RRID: AB_2174164
Purified anti-human CD155 (PVR) (SKII.4)	BioLegend	Cat# 337602; RRID: AB_2300508
HLA class I Bw4 antibody, anti-human, REAfinity™ (REA274)	Miltenyi Biotec	Customized order
HLA class I Bw6 antibody, anti-human, REAfinity™ (REA143)	Miltenyi Biotec	Cat# 130-124-530; RRID: AB_2819663
Purified anti-human CD14 (M5E2)	BioLegend	Cat# 301802; RRID: AB_314184
Purified anti-human CD11b (ICRF44)	BioLegend	Cat# 301302; RRID: AB_314154
Purified anti-human LFA-3 (CD58) TS2/9	BioLegend	Cat# 330902; RRID: AB_1186186
Purified anti-human CD33 (WM53)	BioLegend	Cat# 303402; RRID: AB_314346
Human B7-H6 antibody (875001)	R&D Systems	Cat# MAB7144; RRID: AB_2636810
Purified anti-human CD314 (NKG2D) antibody (1D11)	BioLegend	Cat# 320802; RRID: AB_492956
Purified anti-human CD337 (NKp30) antibody (P30-15)	BioLegend	Cat# 325202; RRID: AB_756106
Purified mouse IgG1, κ isotype control (MOPC-21)	BioLegend	Cat# 400102; RRID: AB_2891079
APC anti-human CD107a (H4A3)	BioLegend	Cat# 328619; RRID: AB_1279057
Brilliant Violet (BV) 711 anti-human CD107a (H4A3)	BioLegend	Cat# 328640; RRID: AB_2565840
APC-H7 mouse anti-human CD107a (H4A3)	BD Biosciences	Cat# 561343; RRID: AB_10644020
PE anti-human CD3 (UCHT1)	BioLegend	Cat# 300441; RRID: AB_2562047
BV421 anti-human CD3 (OKT3)	BioLegend	Cat# 317344; RRID: AB_2565849
BV421 anti-human CD4 (OKT4)	BioLegend	Cat# 317434; RRID: AB_2562134
PE/Cy5 anti-human CD14 (61D3)	ThermoFisher Scientific	Cat# 15-0149-42; RRID: AB_2573058
PE/Cy7 anti-human CD56 (HCD56)	BioLegend	Cat# 318318; RRID: AB_604107
BV605 anti-human CD56 (HCD56)	BioLegend	Cat# 318334; RRID: AB_2562912
Alexa Fluor (AF) 700 anti-human CD16 (3G8)	BioLegend	Cat# 302026; RRID: AB_2278418
PerCP/Cy5.5 anti-human CD16 (3G8)	BioLegend	Cat# 302028; RRID: AB_893262
PerCP/Cy5.5 anti-human CD19 (HIB19)	BioLegend	Cat# 302230; RRID: AB_2073119
PE/Dazzle 594 anti-human CD7 (CD7-6B7)	BioLegend	Cat# 343120; RRID: AB_2650987
APC anti-human CD7 (CD7-6B7)	BioLegend	Cat# 343108; RRID: AB_2291325
APC anti-human B7-H6 (875001)	R&D Systems	Cat# FAB7144A; RRID: AB_3451988
APC anti-human CD48 (BJ40)	BioLegend	Cat# 336713; RRID: AB_2810516
APC anti-human Nectin-2 (TX31)	BioLegend	Cat# 337411; RRID: AB_2565729
APC anti-human LFA-3 (CD58) (TS2/9)	BioLegend	Cat# 330917; RRID: AB_2650885
Human MICA APC-conjugated Antibody (159227)	R&D Systems	Cat# FAB1300A-025; RRID: AB_416836
Human MICB APC-conjugated Antibody (236511)	R&D Systems	Cat# FAB1599A-025; RRID: AB_2297703
Human ULBP-1 APC-conjugated Antibody (170818)	R&D Systems	Cat# FAB1380A; RRID: AB_2923476
Human ULBP-2/5/6 APC-conjugated Antibody (165903)	R&D Systems	Cat# FAB1298A; RRID: AB_2257142
Mouse IgG1 APC-conjugated Antibody (11711)	R&D Systems	Cat# IC002A; RRID: AB_357239
APC Mouse IgG1, κ Isotype Ctrl (Fc) Antibody (MOPC-21)	BioLegend	Cat# 400121; RRID: AB_326443
Mouse IgG2B APC-conjugated Antibody (133303)	R&D Systems	Cat# IC0041A; RRID: AB_357246
Mouse IgG2A APC-conjugated Antibody (20102)	R&D Systems	Cat# IC003A; RRID: AB_357243
V450 mouse anti-human IFN-ɣ (B27)	BD Biosciences	Cat# 560371; RRID: AB_1645594
BV785 anti-human IFN-ɣ (4S.B3)	BioLegend	Cat# 502542; RRID: AB_2563882
BV650 anti-human TNF-ɑ (MAb11)	BioLegend	Cat# 502938; RRID: AB_2562741
FITC anti-HIV p24 (KC57)	Beckman Coulter	Cat# 6604665; RRID: AB_1575987
PE/Cy7 anti-human NKp30 (P30-15)	BioLegend	Cat# 325214; RRID: AB_2716065
PE anti-human NKG2D (1D11)	BioLegend	Cat# 320806; RRID: AB_492960
PE/Dazzle 594 anti-human CD244(2B4) (C1.7)	BioLegend	Cat# 329522; RRID: AB_2572019

**Bacteria and virus strains**		

Replication competent HIV-1 Q23-17	Prepared in this work	N/A
Replication competent HIV-1 THRO 2626	Prepared in this work	N/A
Replication competent HIV-1 CH077 2627	Prepared in this work	N/A
Replication competent HIV-1 CH058 2960	Prepared in this work	N/A
Replication competent HIV-1 CH106 2633	Prepared in this work	N/A
Replication competent HIV-1 NL4-3	Prepared in this work	N/A

**Biological samples**		

LRS chambers	Stanford Blood Center	N/A
Trima residuals	Vitalant Research Institute	N/A

**Chemicals, peptides, and recombinant proteins**		

Anti-human CD28/CD49d antibodies	BD Biosciences	Cat# 347690; RRID: AB_647457
Phytohemagglutinin-L (PHA-L)	ThermoFisher Scientific/eBioscience	00-4977-03
Anti-human CD3 antibody	ThermoFisher Scientific/eBioscience	Cat# 14-0037-82; RRID: AB_467057
Cas9-NLS recombinant protein	UC Berkeley QB3 MacroLab	N/A
Recombinant human IL-2 protein	R&D Systems	202-IL-010
Recombinant Human IL-7 Protein, CF	R&D Systems	BT-007
Recombinant Human IL-15 Protein, CF	R&D Systems	BT-015
Charge altering releasable transporter (CART) O_5_N_5_A_8_	Paul Wender Lab in Stanford University	N/A
Gibco™ PBS (10X), pH 7.4 (for flow cytometry)	Fisher Scientific	70-011-044
Rockland PBS 10X (for CyTOF)	Rockland/VWR	MB-008
Bovine serum albumin (BSA) solution	MilliporeSigma	A9576
UltraPure™ 0.5M EDTA, pH 8.0	ThermoFisher Scientific/Invitrogen	15575020
Sodium azide	MilliporeSigma	71289
Monensin	ThermoFisher Scientific/eBioscience	00-4505-51
Brefeldin A	ThermoFisher Scientific/eBioscience	00-4506-01
Fixable viability dye eFluor 780	ThermoFisher Scientific/eBioscience	65-0865-14
Zombie Aqua fixable viability kit	BioLegend	423101
AViD live/dead fixable yellow dead cell stain kit	ThermoFisher Scientific/Invitrogen	L34959
BD Horizon Brilliant Stain Buffer Plus	BD Biosciences	566385
Human TruStain FcX	BioLegend	422302
BD FACS Lysing solution	BD Biosciences	349202
BD FACS Permeabilizing Solution 2	BD Biosciences	340973
Paraformaldehyde 16% Aqueous Solution EM Grade	Electron Microscopy Sciences	15710
Cisplatin	Enzo Life Sciences	ALX-400-040-M250
Cell-ID™ Intercalator-Ir	Standard BioTools	201192B
DMEM medium, low glucose, pyruvate	ThermoFisher Scientific/Gibco	11885092
RPMI 1640 medium, no glutamine	ThermoFisher Scientific/Gibco	21870092
X-Vivo 15 media	Lonza	02-060Q
Bambanker freezing media	Bulldog Bio	BB02
Fetal bovine serum (FBS)	ThermoFisher Scientific/Gibco	16140071
Fetal bovine serum (FBS)	Corning	35-016-CV
Antibiotic-Antimycotic (100X, containing penicillin, streptomycin, and amphotericin B)	Thermo Fisher Scientific/Gibco	15240062
L-glutamine (200 mM)	Hyclone	SH30034
L-glutamine (200 mM)	Thermo Fisher Scientifc/Gibco	25030081
Dimethyl sulfoxide	MilliporeSigma	D2650
Ficoll-Paque Plus	GE Healthcare	17-1440-03
Fugene 6	Promega	E2691
Sucrose	MilliporeSigma	S0389-500G
ViroMag R/L reagent	OZ Biosciences	RL41000
Gd-157 92%+ Chloride	Trace Sciences International	N/A
In-115 99%+ Chloride	Trace Sciences International	N/A
Y-89 99.9%+ Chloride hexahydrate	Sigma Aldrich	204919
Antibody stabilizer	Candor Bioscience	131 050
Bond-breaker TCEP solution	ThermoFisher Scientific	77720
Raltegravir	BEI Resources	HRP-11680
CellTrace Violet Cell Proliferation Kit	ThermoFisher Scientific	C34557

**Critical commercial assays**		

CD4^+^ T cell isolation kit, human	Miltenyi Biotec	130-096-533
EasySep human CD4^+^ T cell Isolation kit	STEMCELL Technologies	17952
NK cell Isolation kit, human	Miltenyi Biotec	130-092-657
Precision Count Beads	Biolegend	424902
Maxpar® X8 antibody labeling kit, 141Pr	Standard Biotools	201141A
Maxpar® X8 antibody labeling kit, 142Nd	Standard Biotools	201142A
Maxpar® X8 antibody labeling kit, 143Nd	Standard Biotools	201143A
Maxpar® X8 antibody labeling kit, 144Nd	Standard Biotools	201144A
Maxpar® X8 antibody labeling kit, 145Nd	Standard Biotools	201145A
Maxpar® X8 antibody labeling kit, 146Nd	Standard Biotools	201146A
Maxpar® X8 antibody labeling kit, 147Sm	Standard Biotools	201147A
Maxpar® X8 antibody labeling kit, 148Nd	Standard Biotools	201148A
Maxpar® X8 antibody labeling kit, 149Sm	Standard Biotools	201149A
Maxpar® X8 antibody labeling kit, 150Nd	Standard Biotools	201150A
Maxpar® X8 antibody labeling kit, 151Eu	Standard Biotools	201151A
Maxpar® X8 antibody labeling kit, 152Sm	Standard Biotools	201152A
Maxpar® X8 antibody labeling kit, 153Eu	Standard Biotools	201153A
Maxpar® X8 antibody labeling kit, 154Sm	Standard Biotools	201154A
Maxpar® X8 antibody labeling kit, 155Gd	Standard Biotools	201155A
Maxpar® X8 antibody labeling kit, 156Gd	Standard Biotools	201156A
Maxpar® X8 antibody labeling kit, 158Gd	Standard Biotools	201158A
Maxpar® X8 antibody labeling kit, 159Tb	Standard Biotools	201159A
Maxpar® X8 antibody labeling kit, 160Gd	Standard Biotools	201160A
Maxpar® X8 antibody labeling kit, 161Dy	Standard Biotools	201161A
Maxpar® X8 antibody labeling kit, 162Dy	Standard Biotools	201162A
Maxpar® X8 antibody labeling kit, 163Dy	Standard Biotools	201163A
Maxpar® X8 antibody labeling kit, 164Dy	Standard Biotools	201164A
Maxpar® X8 antibody labeling kit, 165Ho	Standard Biotools	201165A
Maxpar® X8 antibody labeling kit, 166Er	Standard Biotools	201166A
Maxpar® X8 antibody labeling kit, 167Er	Standard Biotools	201167A
Maxpar® X8 antibody labeling kit, 168Er	Standard Biotools	201168A
Maxpar® X8 antibody labeling kit, 169Tm	Standard Biotools	201169A
Maxpar® X8 antibody labeling kit, 170Er	Standard Biotools	201170A
Maxpar® X8 antibody labeling kit, 171Yb	Standard Biotools	201171A
Maxpar® X8 antibody labeling kit, 172Yb	Standard Biotools	201172A
Maxpar® X8 antibody labeling kit, 174Yb	Standard Biotools	201174A
Maxpar® X8 antibody labeling kit, 175Lu	Standard Biotools	201175A
Maxpar® X8 antibody labeling kit, 176Yb	Standard Biotools	201176A

**Deposited data**		

Optimization of CD4 T cells and autologous NK cells *in vitro* co-culture system (Figures 1B–1D)	FlowRepository^72^^,^^73^http://flowrepository.org	FlowRepository: FR-FCM-Z7H8
Analysis of the influence of raltegravir treatment on the expression of Gag and downregulation of CD4 in CD4 T cells after HIV infection (Figures S1E–S1G)	Cytobank Community (Beckman Coulter) http://community.cytobank.org	Cytobank Community: 122932
Phenotypic profiling of functional NK cells in response to autologous HIV-infected and mock-infected CD4 T cells (Figures 2 and S2A–S2C)	FlowRepository^72^^,^^73^http://flowrepository.org	FlowRepository: FR-FCM-Z7E5
Analysis of NK cell ligand expression in CD4 T cells that were infected with Q23 strain (Figures 3A–3D and S2D)	FlowRepository^72^^,^^73^http://flowrepository.org	FlowRepository: FR-FCM-Z7H9
Analysis of NK cell ligand expression in CD4 T cells infected with different HIV strains (Figures S3B–S3D)	FlowRepository^72^^,^^73^http://flowrepository.org	FlowRepository: FR-FCM-Z7G8
Analysis of NK cell ligand expression in freshly-isolated, mock-infected and HIV-infected CD4 T cells at 24, 48, and 72 hours post-infection (Figures 3E and S4–S7)	FlowRepository^72^^,^^73^http://flowrepository.org	FlowRepository: FR-FCM-Z8VC
Analysis of NK cell ligand expression and level of HIV infection after CRISPR editing of NK cell ligand-encoding genes (Figures 4B and S9A) and AAVS1 (Figures S8A and S8B)	FlowRepository^72^^,^^73^http://flowrepository.org	FlowRepository: FR-FCM-Z7H7, FR-FCM-Z7H6
Evaluating the function of NK cells co-cultured with HIV-infected and mock-infected CD4 T cells that were edited with CRISPR/Cas9 (Figures 4C and S9B)	FlowRepository^72^^,^^73^http://flowrepository.org	FlowRepository: FR-FCM-Z7H5
Evaluating the expression level of NKp30 and CD244 in CART/mRNA-transfected NK cells (Figures 5B and 5C)	FlowRepository^72^^,^^73^http://flowrepository.org	FlowRepository: FR-FCM-Z7CS
Evaluating the expression level of NKG2D in CART/mRNA-transfected NK cells (Figures 5B, 5C, and S10A–S10C)	FlowRepository^72^^,^^73^http://flowrepository.org	FlowRepository: FR-FCM-Z7CU
Analysis of the function of NK cells that overexpress NKp30, NKG2D, or CD244 in response to HIV-infected, activated mock-infected, and freshly-isolated CD4 T cells (Figures 5D, S10D, and S11)	FlowRepository^72^^,^^73^http://flowrepository.org	FlowRepository: FR-FCM-Z7DB
Analysis of the cytotoxicity of NK cells treated with anti-NKp30 and anti-NKG2D blocking antibody on HIV-infected and mock-infected CD4 T cells (Figures 6B and S12)	Cytobank Community (Beckman Coulter) http://community.cytobank.org	Cytobank Community: 122924

**Experimental models: Cell lines**		

293T	ATCC	CRL-3216
TZM-bl	BEI Resources	ARP-8129

**Oligonucleotides**		

EGFP	TriLink Biotechnologies	L-7601
NKp30	TriLink Biotechnologies	Customized synthesis with DNA template in GenBank: AB055881.1
NKG2D	TriLink Biotechnologies	Customized synthesis with DNA template in Genbank: AF461811.1
DAP10	TriLink Biotechnologies	Customized synthesis with DNA template in Genbank: AF072844.1
CD244 (2B4)	TriLink Biotechnologies	Customized synthesis with DNA template in Genbank: NM_016382.4
See Table S3 for gRNAs used for CRISPR targeting Cas9 crRNA and tracrRNA	Dharmacon Horizon Discovery	N/A

**Recombinant DNA**		

Full length HIV-1 Q23-17	BEI Resources	ARP-12649
Full length HIV-1 pTHRO.c/2626	BEI Resources	HRP-11919/HRP-11745
Full length HIV-1 pCH077.t/2627	BEI Resources	HRP-11919/HRP-11742
Full length HIV-1 pCH058.c/2960	BEI Resources	HRP-11919/HRP-11856
Full length HIV-1 pCH106.c/2633	BEI Resources	HRP-11919/ARP-11743
Full length HIV-1 NL4-3	BEI Resources	ARP-114

**Software and algorithms**		

FlowJo	Tree Star	v10.1 and 10.9
RStudio	R Studio Team	http://www.rstudio.com
BioRender	BioRender.com	N/A
CytoGLMM (R package)	Kronstad et al.^30^; Seiler et al.^38^	https://www.bioconductor.org/packages/CytoGLMM

**Other**		

4D Nucleofector system	Lonza	Lonza AAF-1002B
Ultracentrifuge	Beckman Coulter	Optima XE-90-IVD
3-laser flow cytometer	Miltenyi Biotec	MACSQuant 10
3-laser Aurora spectral cytometer	Cytek Biosciences	Cytek® Aurora
Mass cytometer	Standard BioTools	Helios
Anti-human CD3/CD28 magnetic Dynabeads	ThermoFisher Scientific	11161D
ViroMag magnetic plate	OZ Biosciences	MF10096
EQ Four Element Calibration Beads	Standard BioTools	201078
Amicon Ultra Centrifugal Filter, 30kDa MWCO	Millipore Sigma	UFC503096
Nanosep Centrifugal Devices with Omega Membrane 3K	Pall Corporation	OD003C34
Ultrafree Centrifugal Filter, pore size 0.1 μm	Millipore Sigma	UFC30VV00
LS columns	Miltenyi Biotec	130-042-401
Stericup Quick Release-GP Sterile Vacuum Filtration System	Millipore Sigma	S2GPU10RE
Falcon™ 96-well, non-treated, U-shaped-bottom microplate	Fisher Scientific	08-772-54
Corning® 96-well clear round bottom polypropylene microplate, untreated	VWR	71000-078

### Experimental model and study participant details

#### Cell lines

293T cells were purchased from American Type Culture Collection (ATCC) (catalog number CRL-3216). TZM-bl cells were obtained from BEI Resources (Catalog number ARP-8129). Both 293T and TZM-bl cells were cultured in DMEM media supplemented with 10% FBS (Thermo Fisher) and 1% penicillin/streptomycin/amphotericin (Thermo Fisher).

#### PBMC donors

For CRISPR knockout in CD4 T cells and isolation of autologous NK cells, Trima residuals and leukoreduction system (LRS) chambers were obtained from Vitalant Research Institute (San Francisco, CA) and Stanford Blood Center, and used for isolating peripheral blood mononuclear cells (PBMCs). For other parts of the study, leukoreduction system (LRS) chambers from healthy, de-identified donors were purchased from Stanford Blood Center and used for isolating PBMCs.

### Method details

#### Cell isolation and activation

PBMCs were isolated by density gradient centrifugation using Ficoll-Paque PLUS (GE Healthcare), and cryopreserved in 10% DMSO (Sigma Aldrich) and 90% FBS (Thermo Fisher). For all the assays, PBMCs were thawed, and NK cells and CD4 T cells were separately isolated by negative selection with kits purchased from Miltenyi Biotec unless otherwise indicated. All cells were cultured in RPMI (Gibco), with 10% FBS (Thermo Fisher), 1% L-glutamine (Hyclone) and 1% penicillin/streptomycin/amphotericin (Thermo Fisher) (RP10). NK cells were cultured in RP10 supplemented with 300 IU/ml recombinant human IL-2 (R&D Systems) for 72 hours unless otherwise noted. CD4 T cells were activated in RP10 with plate-bound anti-CD3 (clone OKT3, eBioscience, plates coated at 10 μg/ml), anti-CD28/CD49d (BD Biosciences, final concentration 1 μg/ml per antibody) and PHA-L (eBioscience, final concentration 3.1 μg/ml) for 48 hours in all the experiments unless otherwise noted.

#### Treatment of CD4 T cells with raltegravir

Raltegravir obtained from BEI Resources (HRP-11680) was reconstituted with DMSO to a concentration of 10 mM. CD4 T cells was activated with the method mentioned above for 24 hours and equal volume of RP10 media containing raltegravir (2 μM), anti-CD28/CD49d (BD Biosciences, final concentration 1 μg/ml per antibody) and PHA-L (eBioscience, final concentration 3.1 μg/ml) was added to the CD4 T cell culture. RP10 media containing the same amount of DMSO, anti-CD28/CD49d and PHA-L were added into the control group. Cells were treated for another 24 hours before they were collected and plated for infection or mock-infection.

#### HIV preparation, titration and infection in CD4 T cells

Multiple strains of HIV were prepared by transfecting plasmids encoding full-length, replication competent clones into 293T cells with Fugene 6 (Promega). Plasmids encoding full-length Q23-17 (shortened as Q23), a subtype A strain cloned from a patient one year after seroconversion,^32^ subtype B transmitted/founder (T/F) strains, including THRO 2626, CH077 2627, CH058 2960 and CH106 2633 as well as subtype B laboratory-adapted strain NL4-3 were obtained from BEI Resources. Supernatant from 293T cultures was harvested 48 hours after transfection and viruses were concentrated by ultracentrifugation. Viral stocks were titrated on TZM-bl cells as previously described.^74^ Activated CD4 T cells were infected with HIV using Viromag magnetofection (OZ Biosciences). Magnetofection yields a similar percentage of retrovirus-infected cells compared to spinoculation but allows more efficient and synchronized infection.^36^ Q23 was added into CD4 T cells at a multiplicity of infection (MOI) of 20 in all the experiments except for the analysis of CD4 T cells infected with different HIV strains (Figures S3–S7). To compare how different HIV strains modulate NK cell ligand expression in CD4 T cells, 6 HIV strains, including Q23, THRO 2626, CH077 2627, CH058 2960, CH106 2633, and NL4-3 were added to CD4 T cells at an MOI of 10, 10, 10, 20, 4, and 0.5, respectively, to achieve a similar level of infection. CD4 T cells that were treated with the same amount of Viromag reagent in the absence of HIV (mock-infection) served as a control group. HIV-infected and mock-infected cells were used for co-culture with NK cells, stained with the ligand panel described in Table S2 for mass cytometry (CyTOF) analysis 24 hours post infection, or analyzed for NK cell ligand expression at 24, 48, and 72 hours post infection (hpi). For all the infected or mock-infected cells, unbound viruses or Viromag reagent were not washed out of the culture, until 24, 48 or 72 hpi when they were collected for setting up co-culture or further analysis.

#### CRISPR knockout in CD4 T cells

Trima residuals and leukoreduction system (LRS) chambers were obtained from Vitalant (San Francisco, CA) and Stanford Blood Center, respectively, and PBMCs separated using Ficoll. 3x10^8^ PBMCs were cryopreserved in Bambanker freezing media (Bulldog Bio, catalog number BB02) or in FBS containing 10% DMSO for later NK cell isolation. CD4 T cells were isolated using the EasySep Human CD4 T cell Isolation Kit (STEMCELL Technologies), and activated with anti-CD3/anti-CD28 Dynabeads (Gibco) at a 1:1 bead:cell ratio, in X-Vivo 15 media (Lonza) supplemented with 300 IU/ml IL-2, 5 ng/ml IL-7 and 5 ng/ml IL-15 for 48 hours. Subsequently, activated CD4 T cells were de-beaded, incubated with Cas9 ribonucleoproteins targeting each gene of interest, and electroporated using a 4D Nucleofector system (Lonza) as previously described.^75^ Details of all gRNAs are given in Table S3. Post-electroporation, cells were maintained for 7 days in X-Vivo 15 media supplemented with 300-500 IU/ml IL-2. Cells were then infected with Viromag magnetofection, as described above, with the Q23 virus.

#### Labeling CD4 T cells with CellTrace violet

CD4 T cells were suspended in PBS with 5% FBS at a density of 1x10^7^ cells/ml. CellTrace Violet dye was reconstituted with DMSO at 5 mM and diluted in PBS to a 1 uM working solution. Equal volume of CellTrace Violet working solution was added into suspended CD4 T cells, and CD4 T cells were incubated at room temperature for 20 minutes, before washed with complete RP10 twice. Labeled CD4 T cells were enumerated and co-cultured with NK cells or cultured alone.

#### NK cell transfection with CART

NK cells were isolated from PBMCs of healthy donors as mentioned above. Freshly-isolated NK cells were washed twice with serum-free RPMI (Gibco) media (supplemented with 1% L-glutamine and 1% penicillin/streptomycin/amphotericin), resuspended in serum-free RPMI media and plated in a non-tissue culture treated U-bottom 96-well plate at density of 500,000 NK cells/100 μl in each well for mRNA transfection with CART. mRNA fragments that encode NKp30, NKG2D, DAP10, and CD244 were synthesized at TriLink Biotechnologies with DNA templates of coding sequences (cds) obtained from GenBank® database. During the mRNA synthesis, uridine and cytidine were fully substituted with pseudouridine and 5-methylcytidine. A cap 1 structure (CleanCap AG) as well as a 120-nucleotide polyadenosine (polyA) tail were incorporated into the mRNA fragments to reduce the immunogenicity and enhance the stability of the mRNA. GFP mRNA (catalog number L-7601) was purchased from TriLink Biotechnologies. CARTs are polycationic oligomers that can package polyanionic nucleic acids and deliver the nucleic acids into cells. Once in the cells, CARTs are degraded into nontoxic small molecules and release the nucleic acids.^46^ For co-delivering mRNA that encodes GFP with mRNA that encodes NKp30, NKG2D, CD244, or DAP10, stock of mRNA was premixed to include a combination of NKp30 and GFP mRNA (mass ratio 1:1) or a combination of NKG2D, DAP10, and GFP mRNA (mass ratio 3:1:3 or otherwise as indicated) or a combination of 2B4 and GFP mRNA (mass ratio 1:1) or a combination of DAP10 and GFP mRNA (mass ratio 1:6). The CART/mRNA complexes were prepared by mixing a CART named O_5_:N_5_:A_8_ (shortened as ONA) with each of the premixed mRNA combinations or GFP mRNA alone according to the protocol that was optimized previously and was added into each well of NK cells in the 96-well plate at a dosage of 100 ng mRNA per well.^47^ GFP mRNA that was not mixed with ONA was added into NK cells as an untreated control group for analyzing functional marker expression. Two hours later, media in the 96-well plate was changed to complete RPMI media that contains 10% FBS, 1% L-glutamine and 1% penicillin/streptomycin/amphotericin without adding any cytokine. NK cells were cultured overnight and co-cultured with HIV-infected or mock-infected CD4 T cells.

#### Antibody blockade of NKp30 and NKG2D in NK cells

NK cells were treated with anti-NKp30 (BioLegend, clone P30-15) or anti-NKG2D (BioLegend, clone 1D11) individually or in combination at 10 ug/ml for each antibody^39^^,^^76^, or the corresponding isotype control (BioLegend, mouse IgG1, κ isotype control, clone MOPC-21) of both of these two antibodies at either 10 ug/ml (when each of the blocking antibodies was added individually) or 20 ug/ml (when the blocking antibodies were added in combination) at 37 °C for 2 hours in the presence of 100 IU/ml IL-2 in complete RP10 media. At the end of the treatment, CD4 T cells were directly added into the NK cells for co-culture.

#### CD4 T-NK cell co-culture

NK cells and CD4 T cells were co-cultured for 4 hours, in the presence of brefeldin A (eBioscience, final concentration 3.0 μg/ml), monensin (eBioscience, final concentration 2 μM), and anti-CD107a-APC (for CyTOF analysis in Figure 2), or anti-CD107a-APC-H7 (Figures 1 and 4), or anti-CD107a-BV711 (Figure 5D) at effector:target (E:T) ratio as indicated, except for the analysis of cytotoxicity of NK cells that were treated with antibodies (Figures 6 and S12), where 100 IU/ml IL-2 but not brefeldin A, monensin, or anti-CD107a antibody was added into the co-culture media. At the end of the co-culture, cells were analyzed with flow cytometry or CyTOF.

#### Flow cytometry analysis

Cells were first stained with a viability dye, then stained with antibodies that bind to molecules on the cell surface. For the analysis of NK cell functional markers and killing of HIV-infected CD4 T cells in the *in vitro* co-culture system (Figure 1), cells were first stained with AViD Live/Dead Fixable Yellow Dead Cell Staining Reagent and subsequently stained with PE anti-CD3 (BioLegend, clone UCHT1), PE/Cy7 anti-CD56 (BioLegend, clone HCD56), PerCP/Cy5.5 anti-CD16 (BioLegend, clone 3G8), and APC anti-human CD7 (BioLegend, clone CD7-6B7). For evaluating the purity of NK cells after isolation from PBMCs (Figure S1H), cells were first stained with fixable viability dye eFluor 780, then treated with Human TruStain FcX for blocking Fc receptors and stained with BV421 anti-CD3 (BioLegend, clone OKT3), PE/Dazzle 594 anti-CD7 (BioLegend, clone CD7-6B7), PE/Cy5 anti-CD14 (ThermoFisher Scientific, clone 61D3), Alexa Fluor (AF)700 anti-CD16 (BioLegend, clone 3G8), PerCP/Cy5.5 anti-CD19 (BioLegend, clone HIB19), and PE/Cy7 anti-CD56 (BioLegend, clone HCD56). For the analysis of NK cell ligand expression in CD4 T cells (Figures 3E and S4–S7) or functional analysis of NK cells co-cultured with autologous CRISPR edited CD4 T cells (Figure 4), cells were first stained with Zombie Aqua Fixable Viability dye (Biolegend) and subsequently stained with PE anti-CD3 (BioLegend, clone UCHT1), BV421 anti-CD4 (BioLegend, clone OKT4, not used in the CRISPR experiment as in Figure 4), PE/Cy7 anti-CD56 (BioLegend, clone HCD56, not used for analyzing ligand expression in CD4 T cells) and APC anti-ligand (B7-H6: R&D, clone 875001; CD48: BioLegend, clone; BJ40; Nectin-2: BioLegend, clone TX31; LFA-3: BioLegend, cloneTS2/9; MICA: R&D, clone 159227; MICB: R&D, clone 236511; ULBP1: R&D, clone 170818; ULBP2/5/6: R&D, clone 165903; all ligand antibodies used the same clones as the CyTOF ligand panel in Table S2) and their isotype control antibodies conjugated with APC (IgG1 isotype control from R&D for anti-B7-H6; IgG1 κ isotype control from BioLegend for anti-CD48, anti-Nectin-2, and anti-LFA-3; IgG2B isotype control from R&D for anti-MICA and anti-MICB; IgG2A isotype control from R&D for anti-ULBP1 and anti-ULBP2/5/6) at the same or higher concentration than the corresponding anti-ligand antibody. For the analysis of NKp30, NKG2D, and CD244 expression in NK cells after CART transfection (Figures 5B, 5C, and S10A–S10C), NK cells were first stained with fixable viability dye eFluor 780, then treated with Human TruStain FcX for blocking Fc receptors and stained with Brilliant Violet (BV) 421 anti-CD3 (BioLegend, clone OKT3), APC anti-human CD7 (BioLegend, clone CD7-6B7), BV605 anti-CD56 (BioLegend, clone HCD56), AF700 anti-CD16 (BioLegend, clone 3G8), PE/Cy7 anti-NKp30 (BioLegend, clone P30-15), PE anti-NKG2D (BioLegend, clone 1D11) and PE/Dazzle 594 anti-CD244 (BioLegend, clone C1.7) in the presence of BD Horizon Brilliant Stain Buffer Plus (BD Biosciences). For the functional analysis of CART/mRNA-transfected NK cells in the co-culture with CD4 T cells (Figures 5D, S10D, and S11), cells were first stained with fixable viability dye eFluor 780 and stained with the same panel of antibodies as for analyzing the expression of NKp30, NKG2D and CD244 that was mentioned above, except for APC anti-human CD7. For the analysis of NK cell cytotoxicity on CD4 T cells (Figures 6 and S12), cells were first stained with fixable viability dye eFluor 780, then stained with BV421 anti-CD3 (BioLegend, clone OKT3), PE/Cy7 anti-CD56 (BioLegend, clone HCD56), and AF700 anti-CD16 (BioLegend, clone 3G8).

For the samples that require intracellular staining, cells were subsequently fixed with FACS Lyse (BD Biosciences), permeabilized with FACS Permeabilization Buffer 2 (BD Biosciences), and stained for intracellular markers with V450 anti-IFN-ɣ (BD Biosciences, clone B27) or BV785 anti-IFN-ɣ, BV650 anti-TNF-ɑ (Biolegend, clone MAb11) and FITC anti-HIV p24 (Beckman Coulter, clone KC57) as indicated. Cells were fixed with 1% or 2% paraformaldehyde (PFA) in PBS and analyzed by flow cytometry using an Aurora spectral cytometer (Cytek Biosciences). For the samples that required accurate enumeration of cells, precision count beads (BioLegend, catalog number 424902) of a known concentration were added into the samples and used as a standard to determine the volume of each sample that is acquired by the flow cytometer. Data analysis was performed using FlowJo version 10.1 and 10.9 (Tree Star).

#### Mass cytometry (CyTOF) staining and sample acquisition

All CyTOF antibodies were conjugated using MaxPar X8 labeling kits (Standard BioTools), except for those purchased directly from Standard BioTools; details of all antibodies are given in Table S1 (NK panel) and Table S2 (ligand panel). Table S1 describes a panel of antibodies for analyzing NK cell receptor expression in NK cells after CD4 T-NK cell co-culture (Figure 2). Qdot anti-human CD19, 141Pr anti-human Granzyme B, 142Nd anti-human MIP-1β, 148Nd anti-human LFA-1, 152Sm anti-human Perforin, 167Er anti-human IP-10, and 170Er anti-human KIR2DS2 were not included in data analysis. Table S2 describes a panel of antibodies for analyzing the expression of NK cell ligands on CD4 T cells after HIV-infection or mock-infection (Figures 3A–3D and S3). 144Nd anti-human CD7, 154Sm anti-human CCR2, 166Er anti-human HLA-Bw4, 168Er anti-human HLA-Bw6, 169Tm anti-human CD14, 172Yb anti-human CD33, and 174Yb anti-human CD56 were not included in data analysis. To maintain antibody stability and consistency in staining, all antibody panels were pre-mixed into separate surface and ICS cocktails (as indicated in the Tables), aliquoted and frozen at -80°C until use. At the end of HIV-infection or CD4 T-NK cell co-culture, cells were stained for viability using 25 μM Cisplatin (Enzo) for 1 min and quenched with FBS, and washed with CyFACS buffer (1X Rockland PBS with 0.1% BSA, 2mM EDTA and 0.05% sodium azide). Samples were stained with the surface antibody panel for 30 min at 4°C, fixed with FACS Lyse (BD Biosciences), permeabilized with FACS Permeabilization Buffer 2 (BD Biosciences), and stained with the intracellular staining (ICS) panel for 45 min at 4°C. Cells were suspended in and treated with iridium intercalator (Standard BioTools) in 2% paraformaldehyde (PFA) overnight, and resuspended in 1x EQ Beads (Standard BioTools) before acquisition on a Helios mass cytometer (Standard BioTools).

### Quantification and statistical analysis

#### CyTOF data analysis

Bead normalization (https://github.com/nolanlab/bead-normalization) was performed on all files for each set of experiments post-acquisition. All CyTOF data was visualized and gated using FlowJo v10.1 (Tree Star); gated NK cells (CD3^-^ CD56/CD16^+^), bulk CD4 T cells (CD3^+^ CD8^-^; CD4 was not used for gating as CD4 downregulation can occur after HIV infection) or p24^+/-^ CD4 T cells were exported as fcs files from FlowJo and used in downstream analyses (gating schemes in Figure S2D). The open source statistical software R was used for analyses.^77^

To visualize the multivariate CyTOF data, we used principal components analysis (PCA) on the per sample median marker expression.^78^ We color coded each sample with infection virus strain and blood donor. We used a circle plot to show the correlations of the PCA axes with each marker. To increase interpretability, we focused on markers that have a correlation coefficient of 0.5 or greater along either PCA axes. Additionally, we used a heatmap on the mean asinh-transformed expression with a dendrogram derived from unsupervised clustering.^78^ To improve the visual comparison between markers, we standardized expressions across samples.

To identify phenotypic markers predictive of functionally responding NK cells, HIV-infected (HIV Gag p24^+^) CD4 T cells, or uninfected (p24^-^) CD4 T cells, we used the R package *CytoGLMM* that uses a generalized linear mixed model.^30^^,^^38^ This model takes into account the distribution of each marker and has a donor-specific variable to control for inter-individual variability; significance is based on adjusted p-values by the Benjamini-Hochberg method. For analyses using *CytoGLMM*, raw channel values were transformed using the inverse hyperbolic sine (asinh) function with a cofactor of 5 to account for heteroskedasticity. This transformation was not applied for calculating mean signal intensity values.
