# Supplementary material for: NKp30 and NKG2D contribute to natural killer cell-mediated recognition of HIV-infected cells
Source: iScience. 2025 Sep 12;28(10):113548. doi: 10.1016/j.isci.2025.113548 (PMC12514549; doi:10.1016/j.isci.2025.113548)
Supplement: Document S1. Figures S1–S12 and Tables S1–S3 [file mmc1.pdf]

iScience, Volume *28*

Supplemental information

NKp30 and NKG2D contribute to natural killer cell-mediated recognition of HIV-infected cells

Ruoxi Pi, Nancy Q. Zhao, Allison J. Bien, Thanmayi Ranganath, Christof Seiler, Susan Holmes, Alexander Marson, David N. Nguyen, and Catherine A. Blish

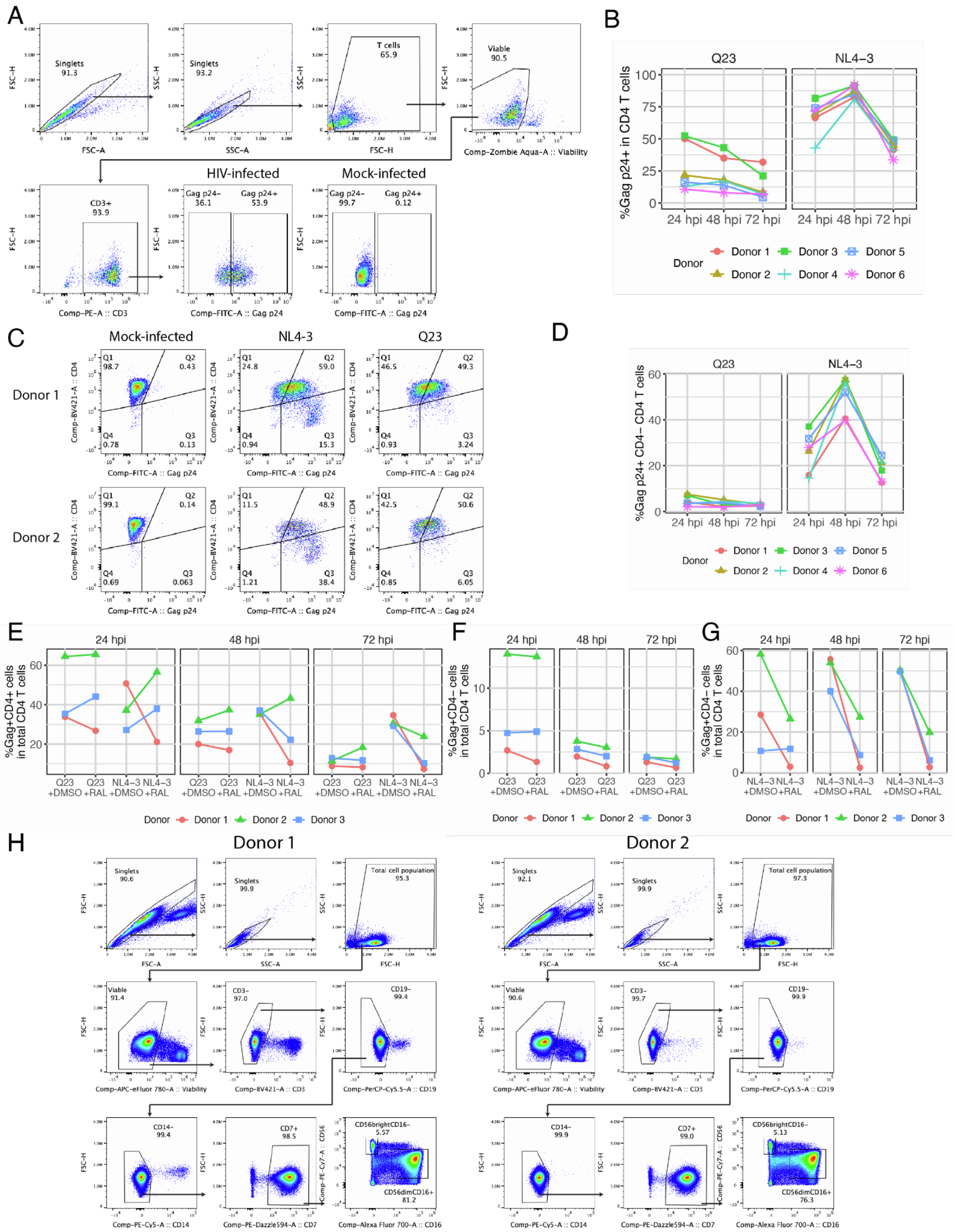
A
Singlets 91.3
Singlets 93.2
T cells 65.9
Viable 90.5
FSC-H
SSC-H
FSC-A
SSC-A
Comp-Zombie Aqua-A :: Viability
HIV-infected
Mock-infected
CD3+ 93.9
Gag p24− 36.1
Gag p24+ 53.9
Gag p24− 99.7
Gag p24+ 0.12
Comp-PE-A :: CD3
Comp-FITC-A :: Gag p24
B
Q23
NL4−3
%Gag p24+ in CD4 T cells
24 hpi 48 hpi 72 hpi
Donor
Donor 1
Donor 2
Donor 3
Donor 4
Donor 5
Donor 6
C
Mock-infected
NL4-3
Q23
Donor 1
Donor 2
Q1 98.7
Q2 0.43
Q4 0.78
Q3 0.13
Q1 24.8
Q2 59.0
Q4 0.94
Q3 15.3
Q1 46.5
Q2 49.3
Q4 0.93
Q3 3.24
Q1 99.1
Q2 0.14
Q4 0.69
Q3 0.063
Q1 11.5
Q2 48.9
Q4 1.21
Q3 38.4
Q1 42.5
Q2 50.6
Q4 0.85
Q3 6.05
Comp-BV421-A :: CD4
Comp-FITC-A :: Gag p24
D
Q23
NL4−3
%Gag p24+ CD4− CD4 T cells
24 hpi 48 hpi 72 hpi
Donor
Donor 1
Donor 2
Donor 3
Donor 4
Donor 5
Donor 6
E
24 hpi
48 hpi
72 hpi
%Gag+CD4+ cells in total CD4 T cells
Q23 +DMSO
Q23 +RAL
NL4−3 +DMSO
NL4−3 +RAL
Donor
Donor 1
Donor 2
Donor 3
F
24 hpi
48 hpi
72 hpi
%Gag+CD4− cells in total CD4 T cells
Q23 +DMSO
Q23 +RAL
G
24 hpi
48 hpi
72 hpi
%Gag+CD4− cells in total CD4 T cells
NL4−3 +DMSO
NL4−3 +RAL
H
Donor 1
Donor 2
Singlets 90.6
Singlets 99.9
Total cell population 95.3
Viable 91.4
CD3− 97.0
CD19− 99.4
CD14− 99.4
CD7+ 98.5
CD56brightCD16− 5.57
CD56dimCD16+ 81.2
Singlets 92.1
Singlets 99.9
Total cell population 97.3
Viable 90.6
CD3− 99.7
CD19− 99.9
CD14− 99.9
CD7+ 99.0
CD56brightCD16− 5.13
CD56dimCD16+ 76.3
Comp-APC-eFluor 780-A :: Viability
Comp-BV421-A :: CD3
Comp-PerCP-Cy5.5-A :: CD19
Comp-PE-Cy5-A :: CD14
Comp-PE-Dazzle594-A :: CD7
Comp-Alexa Fluor 700-A :: CD16
Comp-PE-Cy7-A :: CD56

Figure S1. Identification of human primary CD4 T cells infected with HIV *in vitro* and analysis of NK cells isolated from human peripheral blood mononuclear cells (PBMCs), related to Figure 1.

(A) Representative flow cytometry plots demonstrating the gating strategy to identify Gag $p24^{+}$ cells in HIV-infected CD4 T cells.

(B) Percentage of Gag $p24^{+}$ CD4 T cells at 24, 48 and 72 hours post-infection (hpi) with HIV Q23-17 strain (shortened as Q23, multiplicity of infection, shortened as MOI of 10) and HIV NL4-3 strain (MOI of 0.5) analyzed with flow cytometry. n=6.

(C) Representative flow cytometry plots comparing the downregulation of CD4 in Q23- and NL4-3-infected CD4 T cells.

(D) Percentage of Gag $p24^{+}CD4^{-}$ CD4 T cells at 24, 48 and 72 hpi with Q23 and NL4-3 strains analyzed with flow cytometry. n=6.

(E) Percentage of Gag $p24^{+}$ $CD4^{+}$ T cells in total CD4 T cells at 24, 48 and 72 hpi with Q23 and NL4-3 strains in the presence of raltegravir (+RAL) or DMSO (vehicle of raltegravir) analyzed with flow cytometry. n=3.

(F, G) Percentage of Gag $p24^{+}CD4^{-}$ cells in total CD4 T cells at 24, 48 and 72 hpi with (F) Q23 and (G) NL4-3 strains in the presence of raltegravir (+RAL) or DMSO (vehicle of raltegravir) analyzed with flow cytometry. Scales on y axis for (F) and (G) are different. n=3.

(H) Representative flow cytometry plots of the cells yielded from NK cell isolation from PBMCs of 2 healthy donors demonstrating the percentage of $CD56^{bright}CD16^{-}$ and $CD56^{dim}CD16^{+}$ cells in the NK cell isolates.

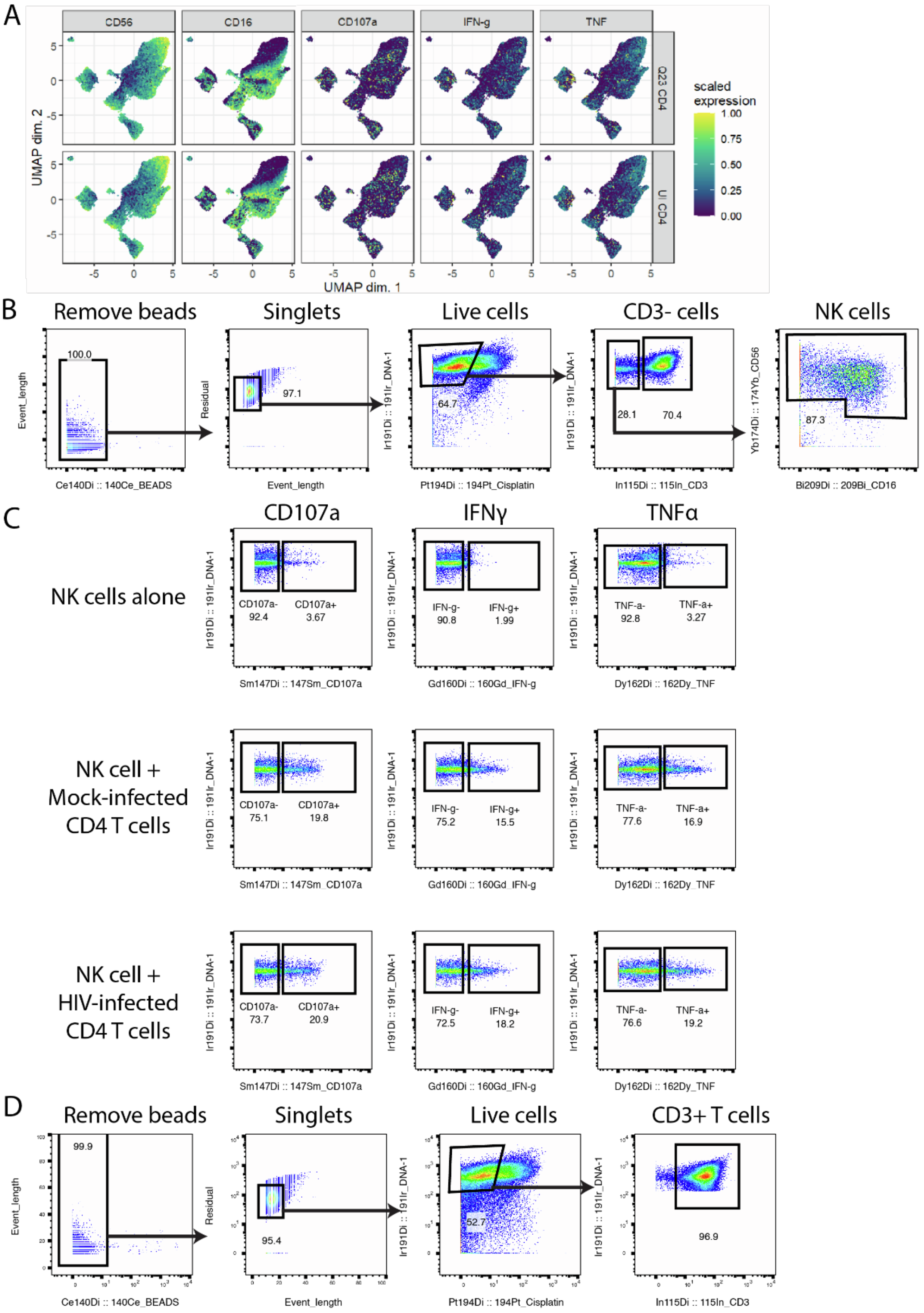
A
CD56
CD16
CD107a
IFN-g
TNF
Q23 CD4
UI CD4
UMAP dim. 2
UMAP dim. 1
scaled expression
1.00
0.75
0.50
0.25
0.00
B
Remove beads
Singlets
Live cells
CD3- cells
NK cells
Event_length
Residual
Ir191Di :: 191Ir_DNA-1
Yb174Di :: 174Yb_CD56
100.0
97.1
64.7
28.1
70.4
87.3
Ce140Di :: 140Ce_BEADS
Event_length
Pt194Di :: 194Pt_Cisplatin
In115Di :: 115In_CD3
Bi209Di :: 209Bi_CD16
C
CD107a
IFNγ
TNFα
NK cells alone
CD107a-
92.4
CD107a+
3.67
IFN-g-
90.8
IFN-g+
1.99
TNF-a-
92.8
TNF-a+
3.27
NK cell +
Mock-infected
CD4 T cells
CD107a-
75.1
CD107a+
19.8
IFN-g-
75.2
IFN-g+
15.5
TNF-a-
77.6
TNF-a+
16.9
NK cell +
HIV-infected
CD4 T cells
CD107a-
73.7
CD107a+
20.9
IFN-g-
72.5
IFN-g+
18.2
TNF-a-
76.6
TNF-a+
19.2
Ir191Di :: 191Ir_DNA-1
Sm147Di :: 147Sm_CD107a
Gd160Di :: 160Gd_IFN-g
Dy162Di :: 162Dy_TNF
D
Remove beads
Singlets
Live cells
CD3+ T cells
Event_length
Residual
Ir191Di :: 191Ir_DNA-1
99.9
95.4
52.7
96.9
Ce140Di :: 140Ce_BEADS
Event_length
Pt194Di :: 194Pt_Cisplatin
In115Di :: 115In_CD3

Figure S2. CyTOF analysis of NK cells co-cultured with HIV-infected or mock-infected autologous CD4 T cells, related to Figures 2 and 3.

(A) Uniform Manifold Approximation and Projection for Dimension Reduction (UMAP) analysis of the expression of markers as labeled on top of each plot in NK cells that were co-cultured with Q23-infected (top row) and mock-infected (bottom row) CD4 T cells.

(B) Gating strategy for NK cells in CyTOF data.

(C) Representative flow cytometry plots of CD107a, IFN-γ, and TNF-α production (by frequency of positive cells), after 4-hour co-culture without (top) or in the presence of mock- (middle) or HIV-infected (bottom) autologous CD4 T cells.

(D) Gating strategy for CD4 T cells in CyTOF data.

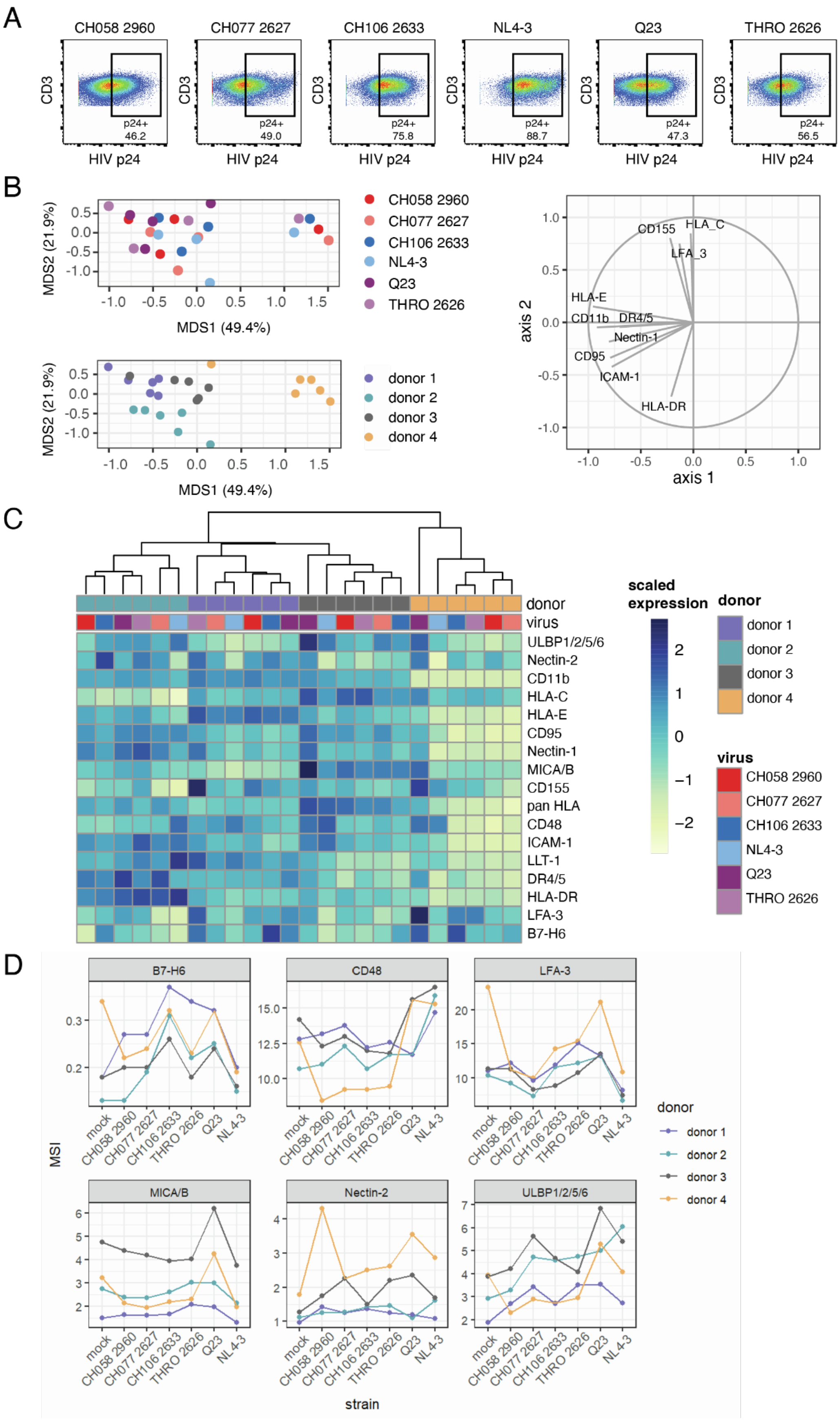

A
CH058 2960
CH077 2627
CH106 2633
NL4-3
Q23
THRO 2626
CD3
HIV p24
p24+ 46.2
p24+ 49.0
p24+ 75.8
p24+ 88.7
p24+ 47.3
p24+ 56.5
B
MDS1 (49.4%)
MDS2 (21.9%)
CH058 2960
CH077 2627
CH106 2633
NL4-3
Q23
THRO 2626
donor 1
donor 2
donor 3
donor 4
axis 1
axis 2
CD155
HLA_C
LFA_3
HLA-E
CD11b
DR4/5
Nectin-1
CD95
ICAM-1
HLA-DR
C
donor
virus
ULBP1/2/5/6
Nectin-2
CD11b
HLA-C
HLA-E
CD95
Nectin-1
MICA/B
CD155
pan HLA
CD48
ICAM-1
LLT-1
DR4/5
HLA-DR
LFA-3
B7-H6
scaled expression
donor
donor 1
donor 2
donor 3
donor 4
virus
CH058 2960
CH077 2627
CH106 2633
NL4-3
Q23
THRO 2626
D
B7-H6
CD48
LFA-3
MICA/B
Nectin-2
ULBP1/2/5/6
MSI
strain
mock
CH058 2960
CH077 2627
CH106 2633
THRO 2626
Q23
NL4-3
donor
donor 1
donor 2
donor 3
donor 4

Figure S3. HIV strain and donor-dependent variability of NK cell ligand modulation in infected CD4 T cells, related to Figure 3.

(A) Representative dot plots from mass cytometry, demonstrating a similar level of infection of primary CD4 T cells with all HIV strains tested, as measured by percentage positively staining for HIV p24.

(B) Principal components analysis (PCA) plot showing separation of all samples, gated to $p24^+$ HIV-infected cells, with each sample coloured by infecting virus strain (top), or blood donor (bottom). Only markers whose contributions are greater than 0.5 in either PCA1 or PCA2 are displayed in the marker loadings.

(C) Dendrogram shows results of unsupervised clustering based on mean asinh-transformed expression of each marker for each sample, gated down to $p24^+$ cells (4 donors, 6 HIV strains in total); heatmap shows scaled mean marker intensity in each sample, scaled within each marker to enable visualization of variation in expression of each NK cell ligand.

(D) Mean signal intensity (MSI) of NK cell ligands on mock-infected CD4 T cells and $p24^+$ CD4 T cells infected with each HIV strain as indicated. n=4. Each individual donor is joined with a line.

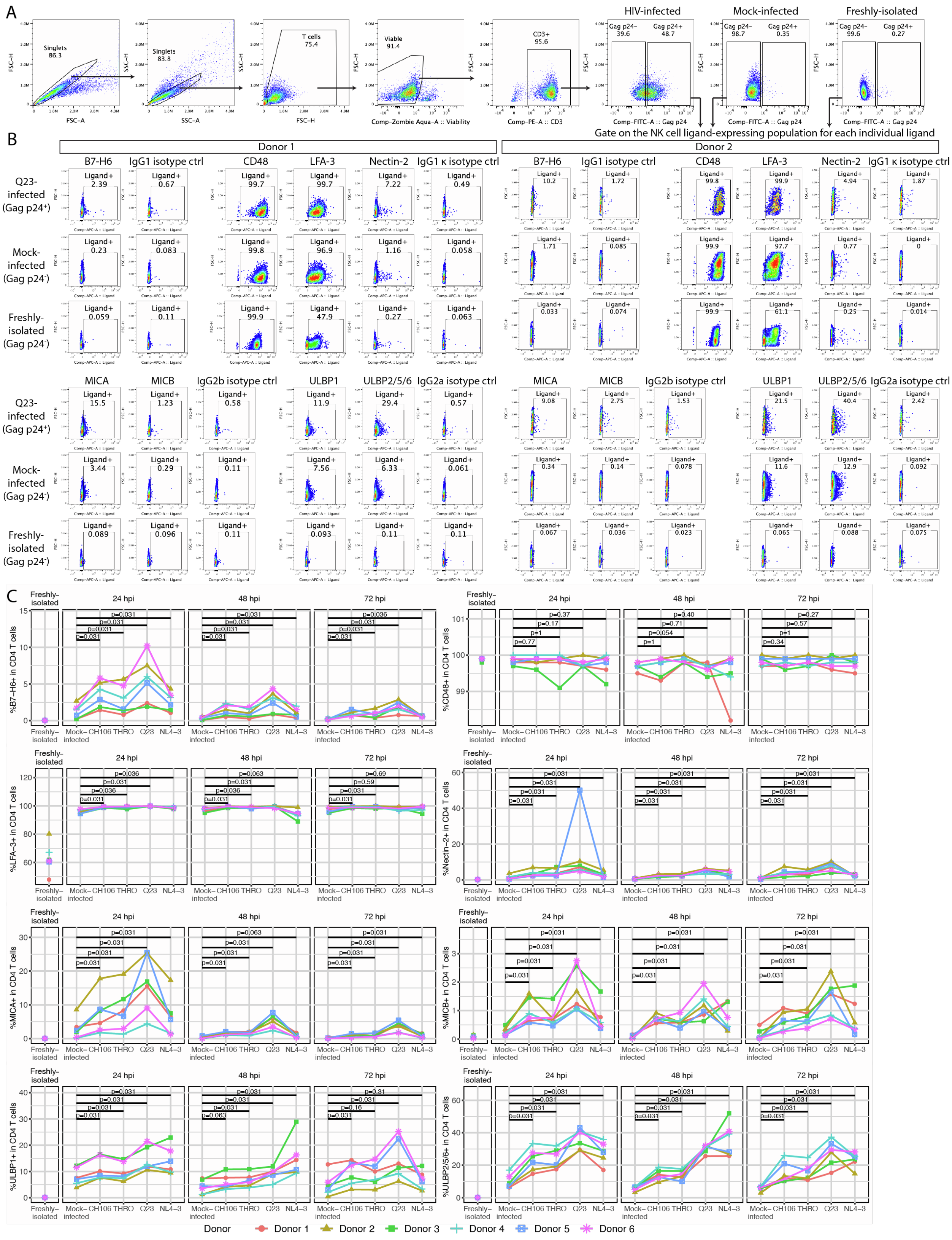
A
HIV-infected
Mock-infected
Freshly-isolated
Gate on the NK cell ligand-expressing population for each individual ligand
B
Donor 1
Donor 2
C
Donor
Donor 1
Donor 2
Donor 3
Donor 4
Donor 5
Donor 6

Figure S4. The expression of NK cell ligands in CD4 T cells at different time points after infection with multiple HIV strains, related to Figure 3E.

(A) Gating strategy to identify HIV-infected (Gag p24$^+$) cells demonstrated with HIV (Q23 strain)-infected (24 hours post-infection), mock-infected and freshly-isolated CD4 T cells. Gag p24$^+$ cells in HIV-infected samples, Gag p24$^-$ cells in mock-infected and freshly-isolated samples were further analyzed for the expression of each NK cell ligand.

(B) Flow cytometry plots demonstrating the gating strategy to identify cells that express each NK cell ligand in HIV (Q23 strain)-infected (Gag p24$^+$), mock-infected (Gag p24$^-$) and freshly-isolated (Gag p24$^-$) CD4 T cells and comparison with the same samples that are stained with the corresponding isotype control antibody (IgG1 isotype control from R&D Systems for B7-H6, IgG1 κ isotype control from BioLegend for CD48, LFA-3, and Nectin-2, IgG2b isotype control from R&D Systems for MICA and MICB, IgG2a isotype control from R&D Systems for ULBP1 and ULBP2/5/6) in 2 donors.

(C) Percentage of cells expressing the NK cell ligand indicated on the y-axis of each panel in CD4 T cells freshly-isolated from PBMCs of healthy donors or at 24, 48, and 72 hours post-infection (hpi) with 4 different HIV strains or with mock-infection. n=6. Statistical analysis was performed with the Wilcoxon signed-rank test.

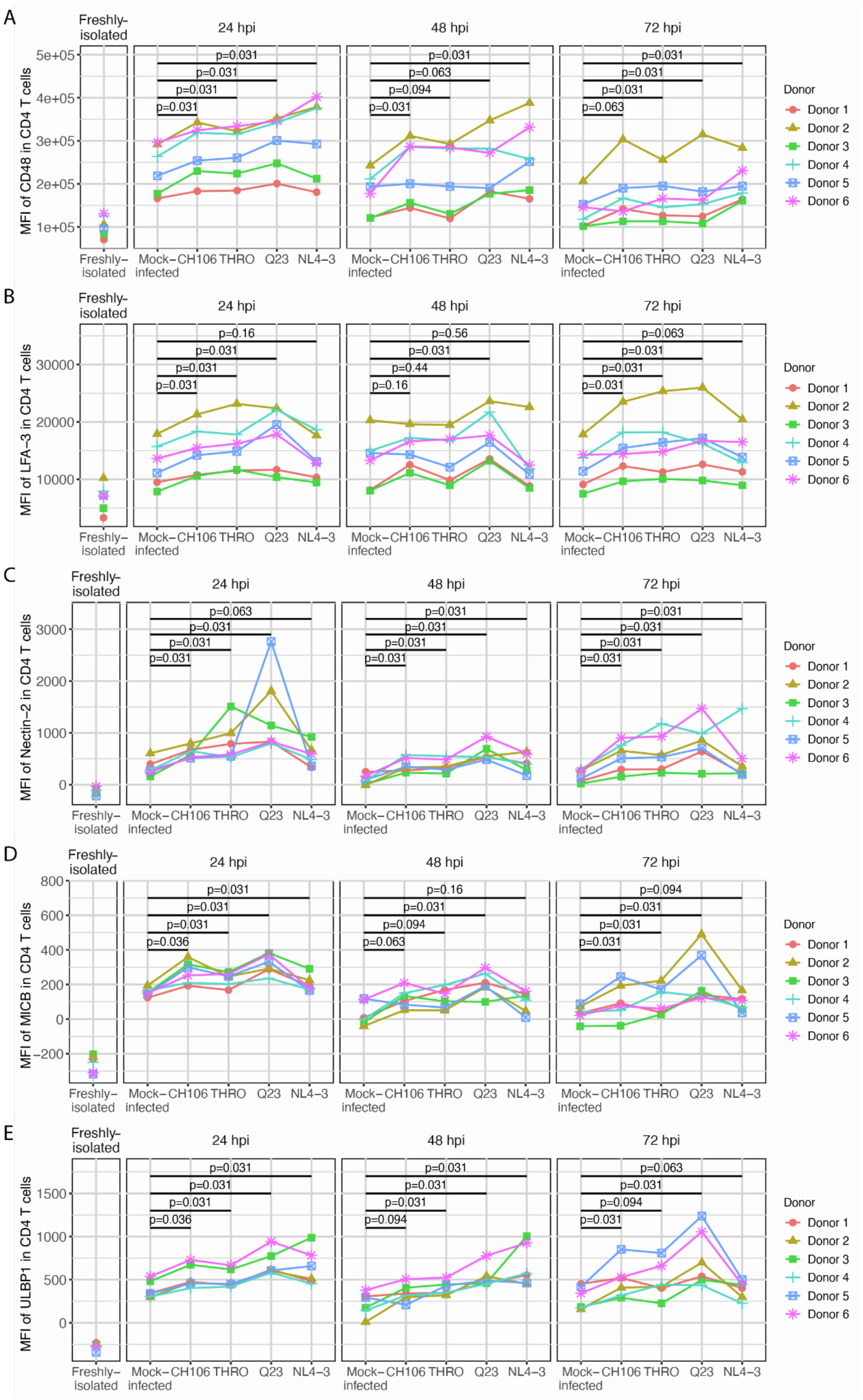
A
Freshly-isolated
24 hpi
48 hpi
72 hpi
MFI of CD48 in CD4 T cells
5e+05
4e+05
3e+05
2e+05
1e+05
p=0.031
p=0.031
p=0.031
p=0.031
p=0.031
p=0.063
p=0.094
p=0.031
p=0.031
p=0.031
p=0.031
p=0.063
Freshly-isolated
Mock-infected
CH106
THRO
Q23
NL4-3
Donor
Donor 1
Donor 2
Donor 3
Donor 4
Donor 5
Donor 6
B
MFI of LFA-3 in CD4 T cells
30000
20000
10000
p=0.16
p=0.031
p=0.031
p=0.031
p=0.56
p=0.031
p=0.44
p=0.16
p=0.063
p=0.031
p=0.031
p=0.031
C
MFI of Nectin-2 in CD4 T cells
3000
2000
1000
0
p=0.063
p=0.031
p=0.031
p=0.031
p=0.031
p=0.031
p=0.031
p=0.031
p=0.031
p=0.031
p=0.031
p=0.031
D
MFI of MICB in CD4 T cells
800
600
400
200
0
-200
p=0.031
p=0.031
p=0.031
p=0.036
p=0.16
p=0.031
p=0.094
p=0.063
p=0.094
p=0.031
p=0.031
p=0.031
E
MFI of ULBP1 in CD4 T cells
1500
1000
500
0
p=0.031
p=0.031
p=0.031
p=0.036
p=0.031
p=0.031
p=0.031
p=0.094
p=0.063
p=0.031
p=0.094
p=0.031

Figure S5. The expression level of NK cell ligands in CD4 T cells at different time points after infection with multiple HIV strains, related to Figure 3E.

Mean fluorescence intensity (MFI) of **(A)** CD48, **(B)** LFA-3, **(C)** Nectin-2, **(D)** MICB, and **(E)** ULBP1 in flow cytometry analysis of CD4 T cells that are freshly-isolated from PBMCs of healthy donors or at 24, 48, and 72 hours post-infection (hpi) with 4 different HIV strains or with mock-infection. n=6. Statistical analysis was performed with the Wilcoxon signed-rank test. Related to Figure 3E.

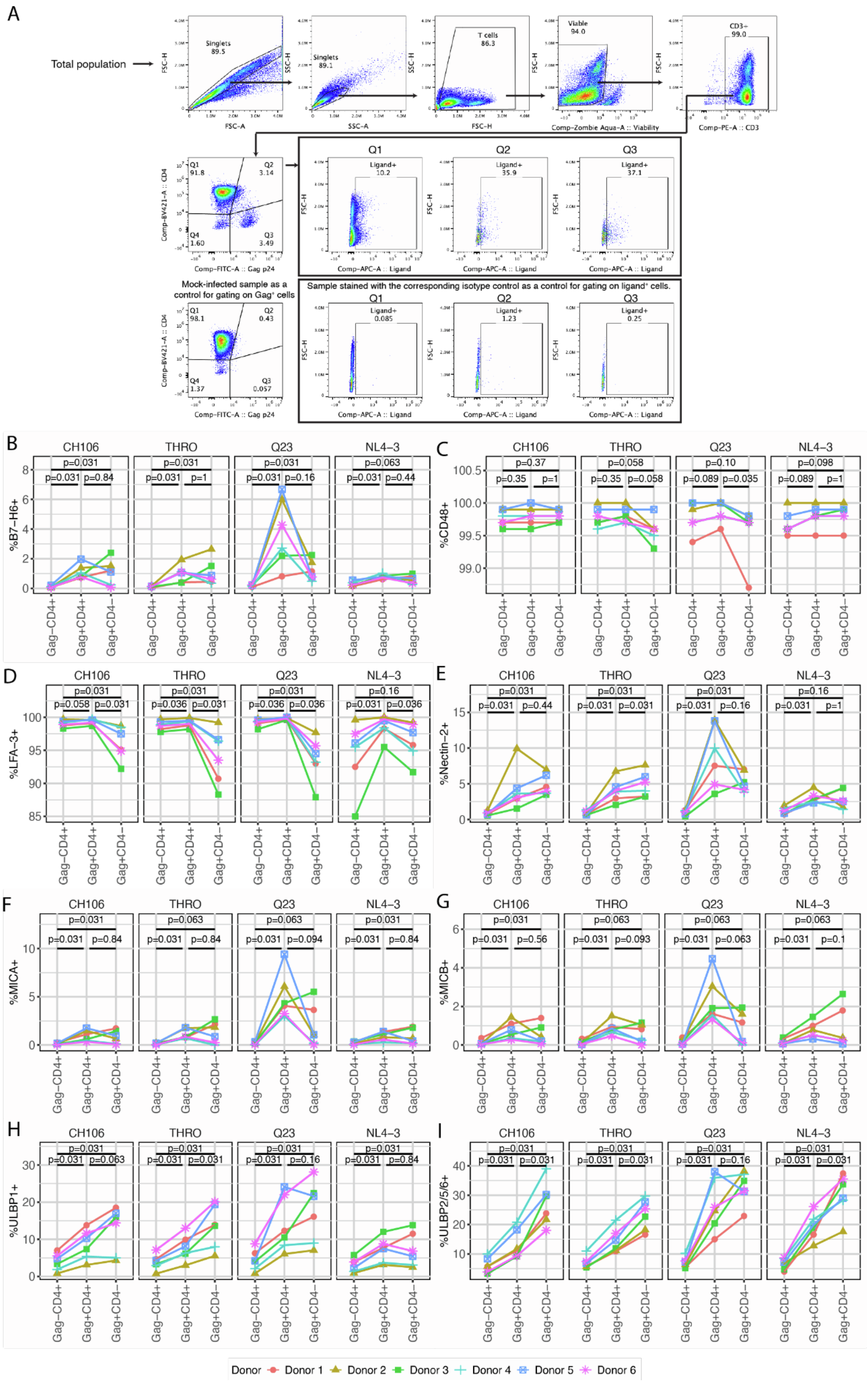

A
Total population
Singlets 89.5
Singlets 89.1
T cells 86.3
Viable 94.0
CD3+ 99.0
Q1 91.8
Q2 3.14
Q4 1.60
Q3 3.49
Q1
Ligand+ 10.2
Q2
Ligand+ 35.9
Q3
Ligand+ 37.1
Mock-infected sample as a control for gating on Gag+ cells
Q1 98.1
Q2 0.43
Q4 1.37
Q3 0.057
Sample stained with the corresponding isotype control as a control for gating on ligand+ cells.
Ligand+ 0.085
Ligand+ 1.23
Ligand+ 0.25
Comp-FITC-A :: Gag p24
Comp-BV421-A :: CD4
Comp-APC-A :: Ligand
Comp-Zombie Aqua-A :: Viability
Comp-PE-A :: CD3
B
%B7–H6+
C
%CD48+
D
%LFA–3+
E
%Nectin–2+
F
%MICA+
G
%MICB+
H
%ULBP1+
I
%ULBP2/5/6+
CH106
THRO
Q23
NL4–3
Gag–CD4+
Gag+CD4+
Gag+CD4–
Donor
Donor 1
Donor 2
Donor 3
Donor 4
Donor 5
Donor 6

Figure S6. Percentage of HIV-infected cells that express NK cell ligands at the stage of pre- and post-integration at 72 hours post-infection, related to Figure 3E.

(A) Gating strategy in flow cytometry analysis to identify HIV-infected cells at the stage of pre-integration (Gag p24^{+}CD4^{+} in Q2), post-integration (Gag p24^{+}CD4^{-} in Q3) and bystanders (Gag^{-}CD4^{+} in Q1), and to identify the cells that express NK cell ligands in these 3 populations. The representative plots are from a sample infected with Q23.17 strain and stained with APC anti-ULBP2/5/6 antibody. The same sample was stained with the IgG2a isotype control of APC anti-ULBP2/5/6 antibody and serves as a negative control to gate on cells that are ULBP2/5/6^{+}. A sample with mock-infected cells analyzed with the same gating strategy in parallel serves as a negative control to gate on cells that are Gag p24^{+}.

(B-I) Percentage of cells in Gag p24^{-}CD4^{+} (bystanders, labeled as Gag^{-}CD4^{+}), Gag p24^{+}CD4^{+} (pre-integration, labeled as Gag^{+}CD4^{+}), and Gag p24^{+}CD4^{-} (post-integration, labeled as Gag^{+}CD4^{-}) populations that express (B) B7-H6, (C) CD48, (D) LFA-3 (CD58), (E) Nectin-2 (CD112), (F) MICA, (G) MICB, (H) ULBP1, (I) ULBP2/5/6 at 72 hours post-infection with the HIV strain labeled on the top of each plot. n=6. Statistical analysis was performed with the Wilcoxon signed-rank test.

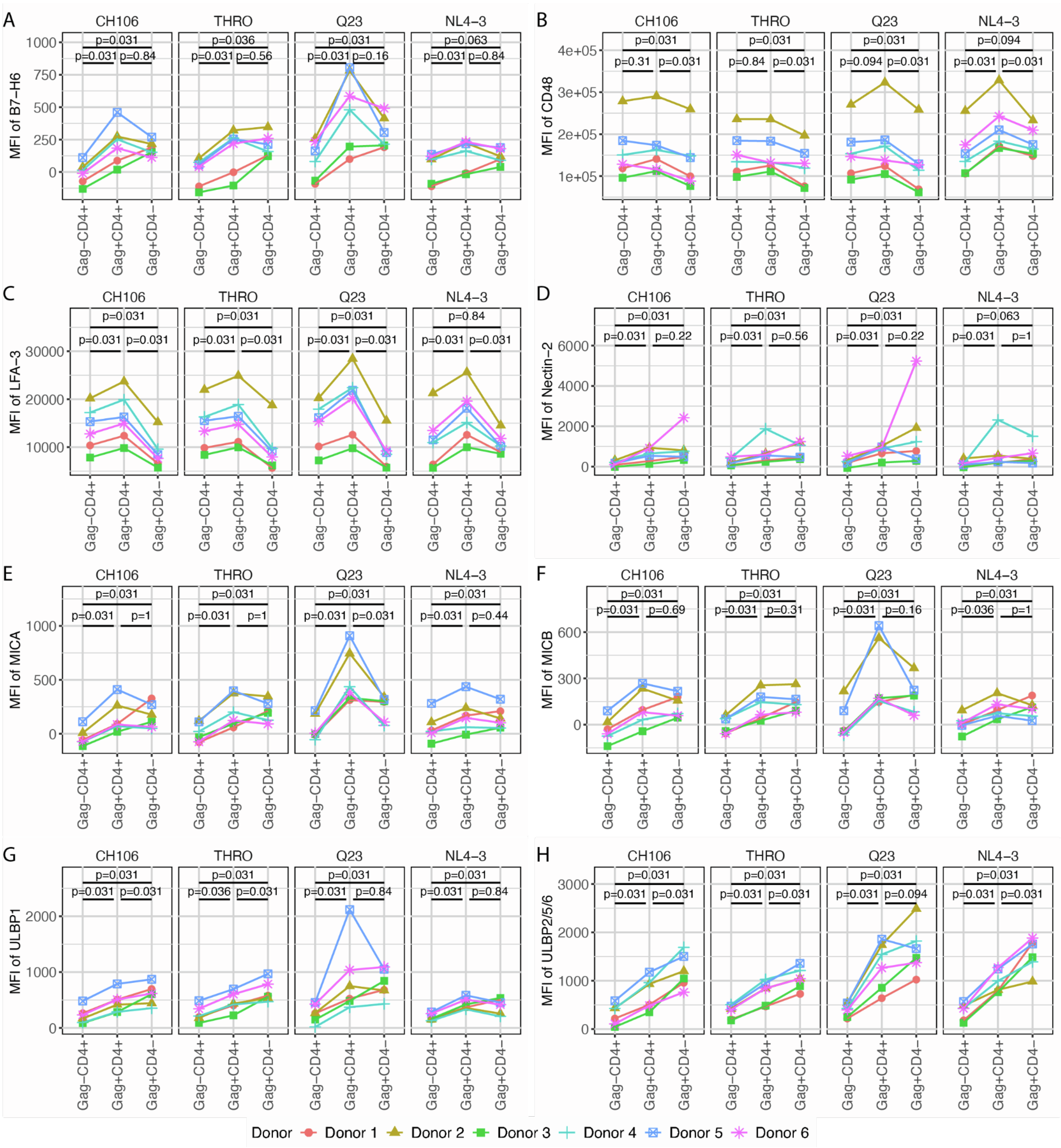

Figure S7. Expression level of NK cell ligands in HIV-infected cells at the stage of pre- and post-integration at 72 hours post-infection, related to Figure 3E. Mean fluorescence intensity of **(A)** B7-H6, **(B)** CD48, **(C)** LFA-3 (CD58), **(D)** Nectin-2 (CD112), **(E)** MICA, **(F)** MICB, **(G)** ULBP1, **(H)** ULBP2/5/6 indicating the expression level of the corresponding NK cell ligands in Gag p24$^-$CD4$^+$ (bystanders, labeled as Gag$^-$CD4$^+$), Gag p24$^+$CD4$^+$ (pre-integration, labeled as Gag$^+$CD4$^+$), and Gag p24$^+$CD4$^-$ (post-integration, labeled as Gag$^+$CD4$^-$) populations at 72 hours post-infection with the HIV strain indicated on the top of each plot. n=6. Statistical analysis was performed with the Wilcoxon signed-rank test.

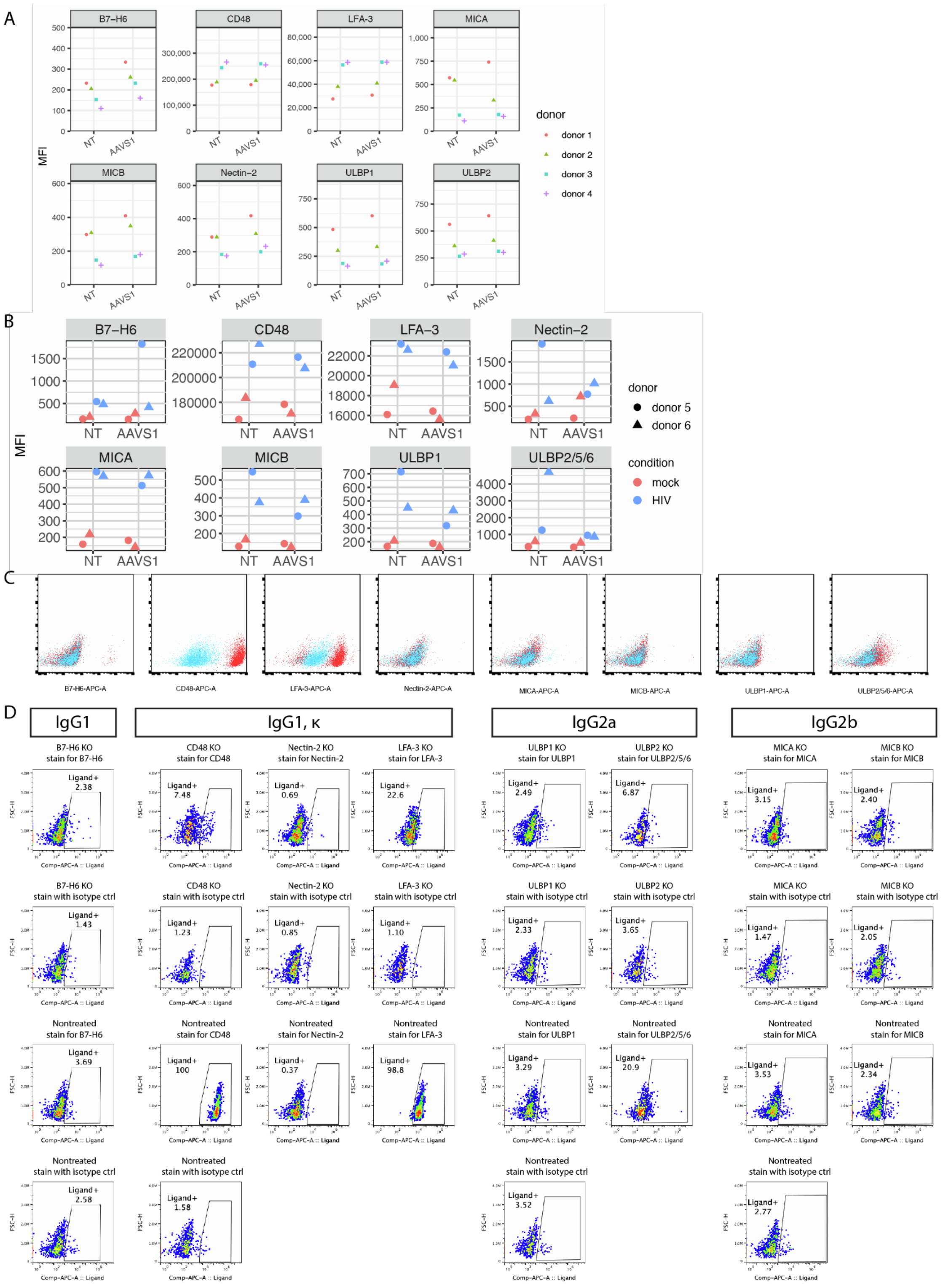
A
B7−H6
CD48
LFA-3
MICA
MICB
Nectin−2
ULBP1
ULBP2
MFI
NT
AAVS1
donor
donor 1
donor 2
donor 3
donor 4
B
B7−H6
CD48
LFA−3
Nectin−2
MICA
MICB
ULBP1
ULBP2/5/6
MFI
NT
AAVS1
donor
donor 5
donor 6
condition
mock
HIV
C
B7-H6-APC-A
CD48-APC-A
LFA-3-APC-A
Nectin-2-APC-A
MICA-APC-A
MICB-APC-A
ULBP1-APC-A
ULBP2/5/6-APC-A
D
IgG1
IgG1, κ
IgG2a
IgG2b
B7-H6 KO stain for B7-H6
Ligand+ 2.38
CD48 KO stain for CD48
Ligand+ 7.48
Nectin-2 KO stain for Nectin-2
Ligand+ 0.69
LFA-3 KO stain for LFA-3
Ligand+ 22.6
ULBP1 KO stain for ULBP1
Ligand+ 2.49
ULBP2 KO stain for ULBP2/5/6
Ligand+ 6.87
MICA KO stain for MICA
Ligand+ 3.15
MICB KO stain for MICB
Ligand+ 2.40
B7-H6 KO stain with isotype ctrl
Ligand+ 1.43
CD48 KO stain with isotype ctrl
Ligand+ 1.23
Nectin-2 KO stain with isotype ctrl
Ligand+ 0.85
LFA-3 KO stain with isotype ctrl
Ligand+ 1.10
ULBP1 KO stain with isotype ctrl
Ligand+ 2.33
ULBP2 KO stain with isotype ctrl
Ligand+ 3.65
MICA KO stain with isotype ctrl
Ligand+ 1.47
MICB KO stain with isotype ctrl
Ligand+ 2.05
Nontreated stain for B7-H6
Ligand+ 3.69
Nontreated stain for CD48
Ligand+ 100
Nontreated stain for Nectin-2
Ligand+ 0.37
Nontreated stain for LFA-3
Ligand+ 98.8
Nontreated stain for ULBP1
Ligand+ 3.29
Nontreated stain for ULBP2/5/6
Ligand+ 20.9
Nontreated stain for MICA
Ligand+ 3.53
Nontreated stain for MICB
Ligand+ 2.34
Nontreated stain with isotype ctrl
Ligand+ 2.58
Nontreated stain with isotype ctrl
Ligand+ 1.58
Nontreated stain with isotype ctrl
Ligand+ 3.52
Nontreated stain with isotype ctrl
Ligand+ 2.77
FSC-H
Comp-APC-A :: Ligand

Figure S8. CRISPR knockout of individual genes that encode NK cell ligands in CD4 T cells, related to Figure 4.
(A) NK cell ligand expression in untreated and AAVS1 controls. Mean fluorescence intensity (MFI) of 8 targeted NK cell ligands, in non-treated (NT) and AAVS1 KO (AAVS1) controls. Each donor is indicated by a different symbol. n=4.
(B) NK cell ligand expression in mock-infected and HIV-infected (Gag p24$^+$) CD4 T cells in untreated and AAVS1 controls. Mean fluorescence intensity (MFI) of 8 targeted NK cell ligands, in non-treated (NT) and AAVS1 edited (AAVS1) controls from 2 donors other than the donors in (A). Each donor is indicated by a different symbol.
(C) Representative flow cytometry plots comparing the expression level of NK cell ligands in CD4 T cells from donor 2 with CRISPR knockout of each individual ligand (in blue) or without treatment (NT, in red).
(D) Representative flow cytometry plots comparing the staining with ligand-specific antibodies and the corresponding isotype control antibodies in CD4 T cells with individual gene knockout and nontreated CD4 T cells. The isotype of antibodies labeled in the rectangles above the plots indicate the isotype of all the ligand-specific antibodies and the corresponding isotype controls.

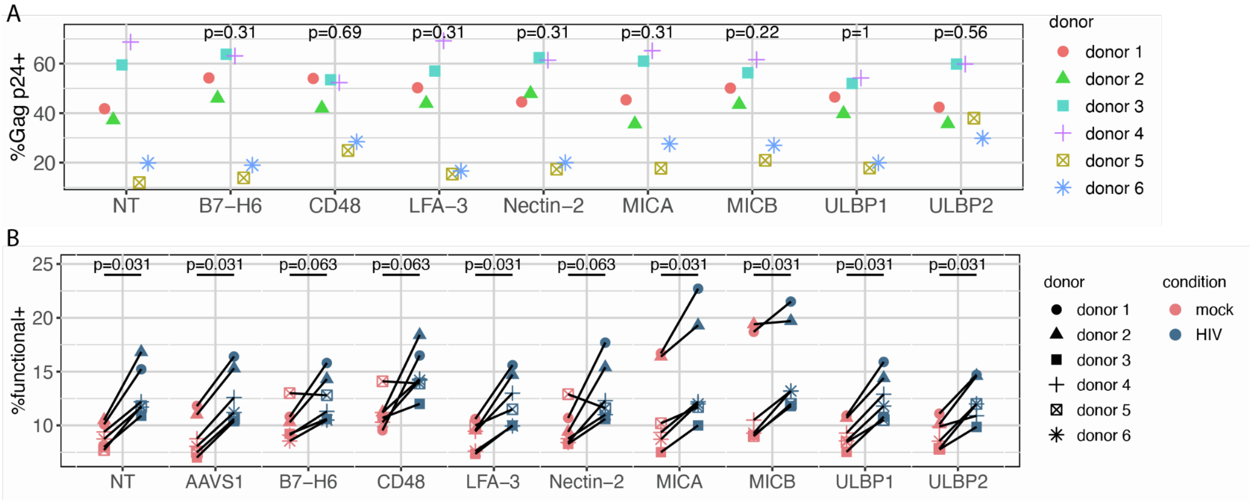

Figure S9. Evaluating the influence of functional response of NK cells to CD4 T cells with knockout of individual genes that encode NK cell ligands, related to Figure 4.

(A) Percentage of Gag p24⁺ cells in CD4 T cells that are not treated or with knockout of individual NK cell ligands. n=6. Each donor is indicated by a different symbol. Statistical analysis was performed with the Wilcoxon signed-rank test.

(B) Percentage of functional⁺ NK cells (cells that were stained positive for any of CD107a, IFN-γ, and TNF-α) indicating NK cell responses to mock- (in pink) and HIV-infected (in blue) CD4 T cells, in all knockout conditions or NT control after NK cells and CD4 T cells were co-cultured at effector:target ratio of 1:1. n=6. Each donor is indicated by a different symbol. Statistical analysis was performed with the Wilcoxon signed-rank test.

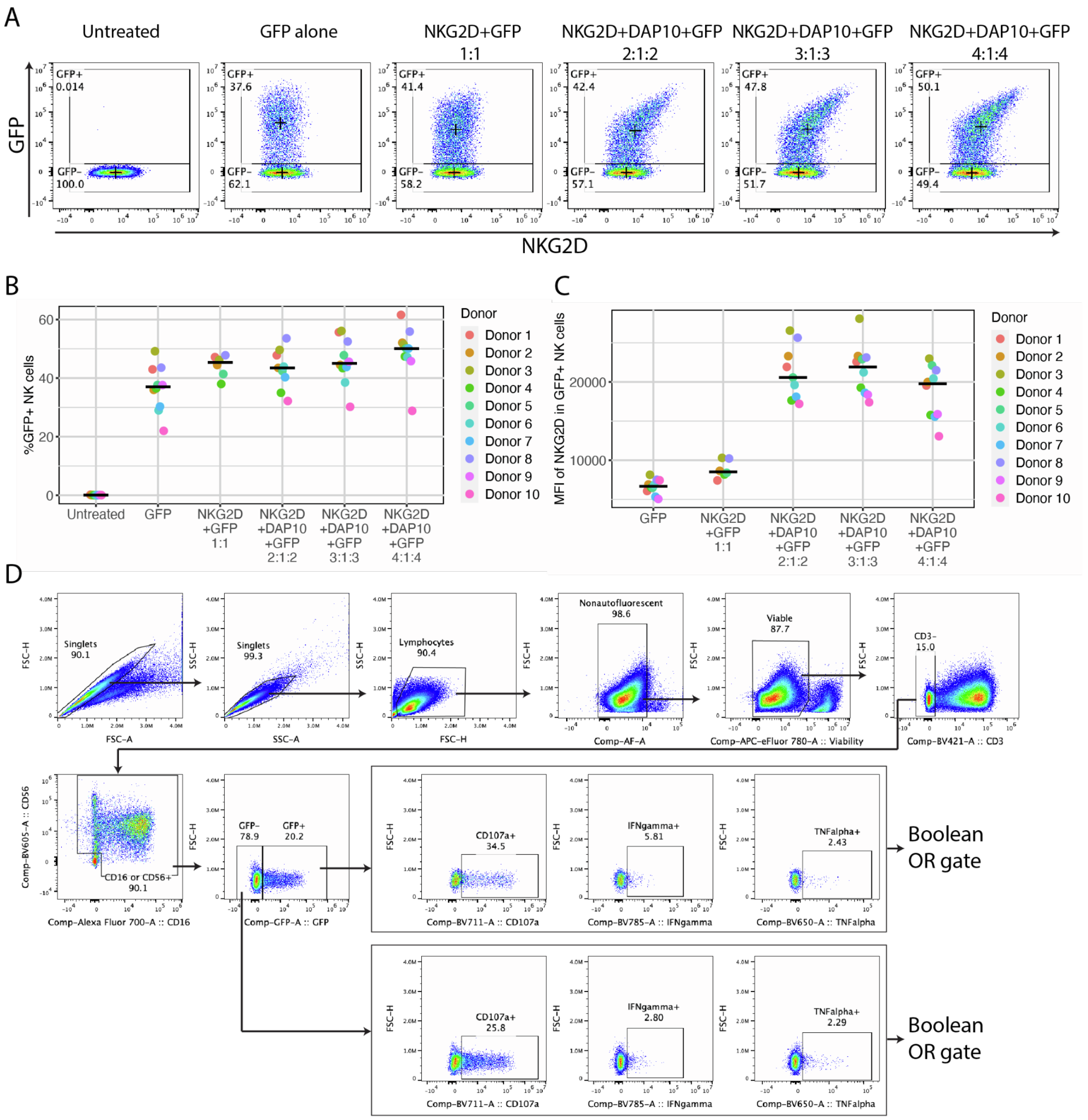

Figure S10. Co-transfection of NKG2D, DAP10, and GFP mRNA in primary NK cells with CART and gating strategy to identify functional NK cells, related to Figure 5.

(A) Representative flow cytometry plots indicating the co-expression of GFP and NKG2D in NK cells purified from PBMCs of healthy donors after the NK cells were transfected with the encoding mRNA according to the ratios as indicated on top of each plot. Crosses indicate the median fluorescence intensity of the marker on x and y axes in each gate.

(B) Percentage of NK cells that express GFP after the cells were transfected with the encoding mRNA according to the ratios labeled on x axis. Bars indicate the median in each group. n=6-10.

(C) Mean fluorescence intensity (MFI) of anti-human NKG2D-PE signal indicating the expression level of NKG2D in GFP$^+$ NK cells after NK cells were transfected with the encoding mRNA according to the ratios labeled on x axis. Bars indicate the median in each group. n=6-10.

(D) Gating strategy of identifying the transfected (GFP$^+$) and untransfected (GFP$^-$) NK cells in NK-CD4 T cells co-culture assay as shown in Figure 5D.

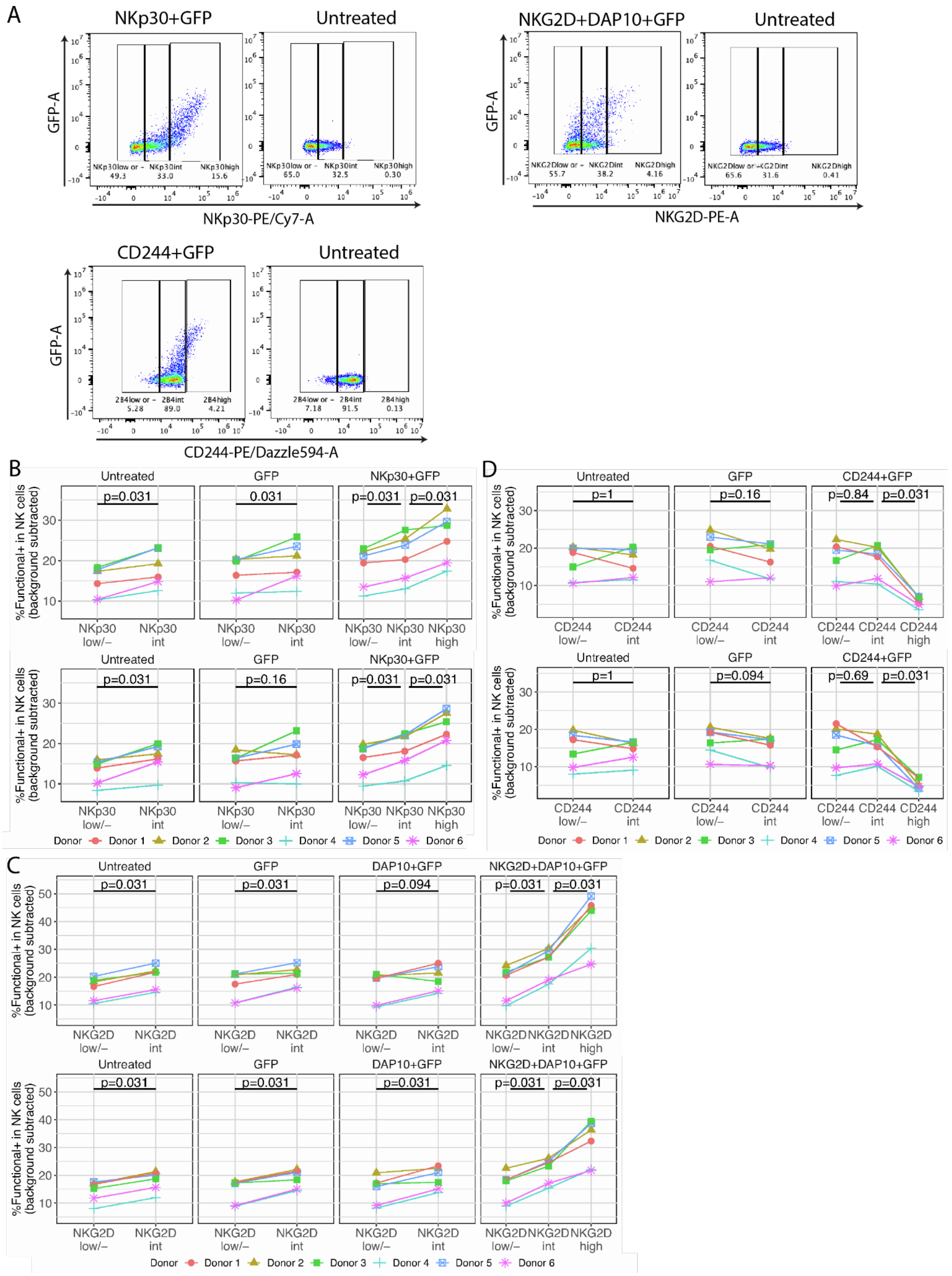
A
NKp30+GFP
Untreated
NKG2D+DAP10+GFP
Untreated
GFP-A
NKp30low or – 49.3
NKp30int 33.0
NKp30high 15.6
NKp30low or – 65.0
NKp30int 32.5
NKp30high 0.30
NKp30-PE/Cy7-A
NKG2Dlow or – 55.7
NKG2Dint 38.2
NKG2Dhigh 4.16
NKG2Dlow or – 65.6
NKG2Dint 31.6
NKG2Dhigh 0.41
NKG2D-PE-A
CD244+GFP
Untreated
2B4low or – 5.28
2B4int 89.0
2B4high 4.21
2B4low or – 7.18
2B4int 91.5
2B4high 0.13
CD244-PE/Dazzle594-A
B
Untreated
GFP
NKp30+GFP
p=0.031
0.031
p=0.031
p=0.031
p=0.031
p=0.16
p=0.031
p=0.031
%Functional+ in NK cells (background subtracted)
NKp30 low/–
NKp30 int
NKp30 high
Donor
Donor 1
Donor 2
Donor 3
Donor 4
Donor 5
Donor 6
D
Untreated
GFP
CD244+GFP
p=1
p=0.16
p=0.84
p=0.031
p=1
p=0.094
p=0.69
p=0.031
CD244 low/–
CD244 int
CD244 high
C
Untreated
GFP
DAP10+GFP
NKG2D+DAP10+GFP
p=0.031
p=0.031
p=0.094
p=0.031
p=0.031
p=0.031
p=0.031
p=0.031
p=0.031
p=0.031
NKG2D low/–
NKG2D int
NKG2D high

Figure S11. Correlation between NK cell response to CD4 T cells and expression level of NKp30, NKG2D and CD244, related to Figure 5.

(A) Representative flow cytometry plots indicating the gating strategy to stratify NK cells into populations that express high, intermediate (int), or low/negative level of NKp30, NKG2D, or CD244 in NK cells that were co-transfected with the mRNA as labeled or in untreated NK cells.

(B-D) Percentage of functional^{+} NK cells (positive for any of CD107a, IFN-ɣ, and TNF-ɑ) in NK cell populations that express high, intermediate (int) or low/negative level of (B) NKp30, (C) NKG2D, and (D) CD244 after transfected with the mRNA as labeled on top of the panels and co-cultured with autologous HIV-infected CD4 T cells (top panel of (B),(C) and (D)) and mock-infected CD4 T cells (bottom panel of (B), (C), and (D)) after subtracting the percentage of functional^{+} NK cells in NK alone group (background subtraction). n=6 in (B), (C), and (D). Statistical analysis was performed with the Wilcoxon signed-rank test in (B), (C) and (D).

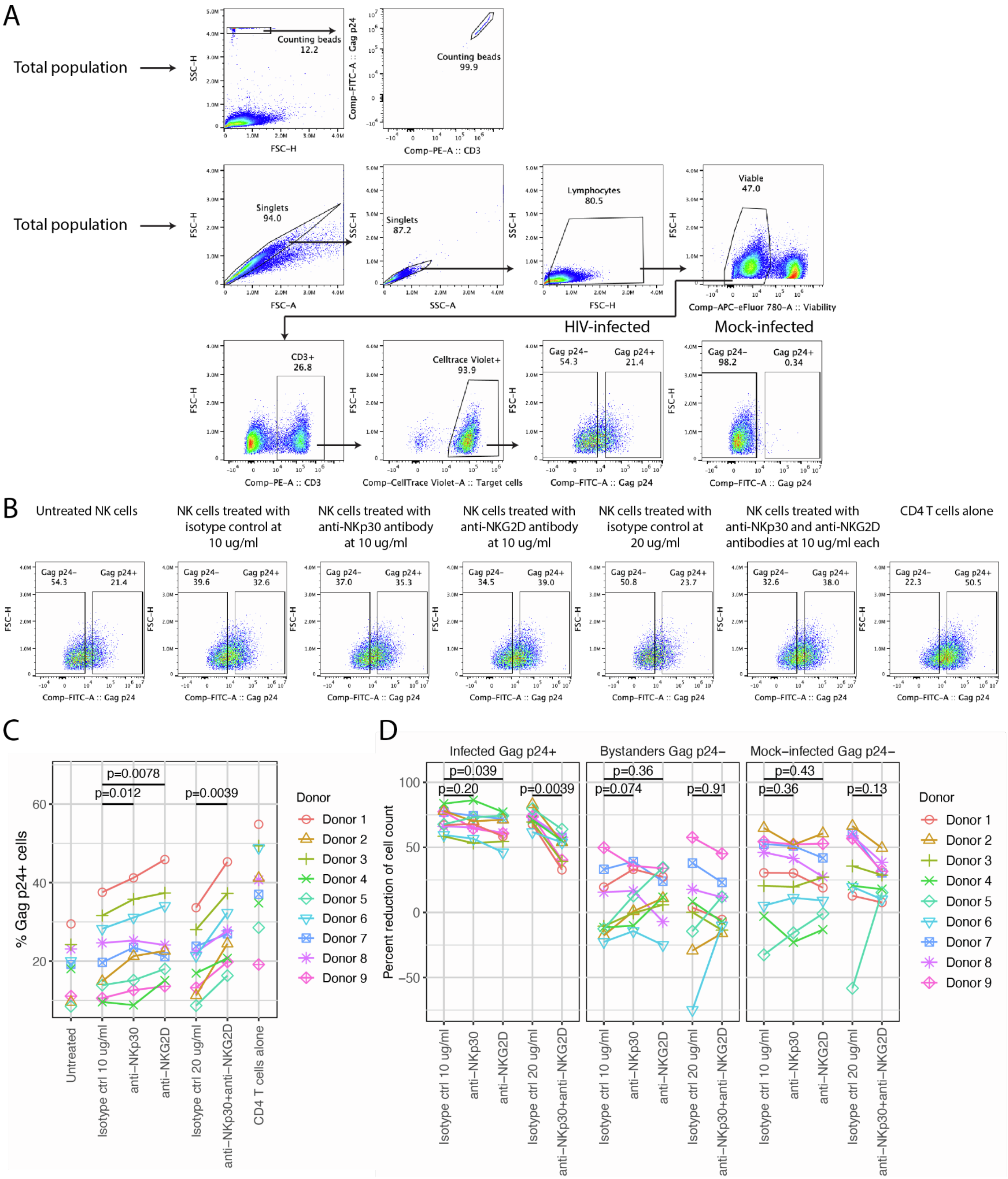

A
Total population
Counting beads 12.2
SSC-H
FSC-H
Comp-FITC-A :: Gag p24
Counting beads 99.9
Comp-PE-A :: CD3
Total population
Singlets 94.0
FSC-A
Singlets 87.2
SSC-A
Lymphocytes 80.5
Viable 47.0
Comp-APC-eFluor 780-A :: Viability
HIV-infected
Mock-infected
CD3+ 26.8
Comp-PE-A :: CD3
Celltrace Violet+ 93.9
Comp-CellTrace Violet-A :: Target cells
Gag p24− 54.3
Gag p24+ 21.4
Gag p24− 98.2
Gag p24+ 0.34
Comp-FITC-A :: Gag p24
B
Untreated NK cells
NK cells treated with isotype control at 10 ug/ml
NK cells treated with anti-NKp30 antibody at 10 ug/ml
NK cells treated with anti-NKG2D antibody at 10 ug/ml
NK cells treated with isotype control at 20 ug/ml
NK cells treated with anti-NKp30 and anti-NKG2D antibodies at 10 ug/ml each
CD4 T cells alone
Gag p24− 54.3
Gag p24+ 21.4
Gag p24− 39.6
Gag p24+ 32.6
Gag p24− 37.0
Gag p24+ 35.3
Gag p24− 34.5
Gag p24+ 39.0
Gag p24− 50.8
Gag p24+ 23.7
Gag p24− 32.6
Gag p24+ 38.0
Gag p24− 22.3
Gag p24+ 50.5
C
% Gag p24+ cells
p=0.0078
p=0.012
p=0.0039
Untreated
Isotype ctrl 10 ug/ml
anti−NKp30
anti−NKG2D
Isotype ctrl 20 ug/ml
anti−NKp30+anti−NKG2D
CD4 T cells alone
Donor
Donor 1
Donor 2
Donor 3
Donor 4
Donor 5
Donor 6
Donor 7
Donor 8
Donor 9
D
Percent reduction of cell count
Infected Gag p24+
Bystanders Gag p24−
Mock−infected Gag p24−
p=0.039
p=0.20
p=0.0039
p=0.36
p=0.074
p=0.91
p=0.43
p=0.36
p=0.13
Isotype ctrl 10 ug/ml
anti−NKp30
anti−NKG2D
Isotype ctrl 20 ug/ml
anti−NKp30+anti−NKG2D
Donor
Donor 1
Donor 2
Donor 3
Donor 4
Donor 5
Donor 6
Donor 7
Donor 8
Donor 9

Figure S12. Evaluating NK cell cytotoxicity on HIV-infected, bystander, and mock-infected CD4 T cells, related to Figure 6.
(A) Representative flow cytometry plots indicating the gating strategy to identify Gag p24$^+$ CD4 T cells and bystander (Gag p24$^-$) CD4 T cells in HIV-infected groups and Gag p24$^-$ CD4 T cells in mock-infected groups.
(B) Representative flow cytometry plots comparing the percentage of Gag p24$^+$ in CD4 T cells after co-cultured with NK cells treated with each condition as indicated above each plot.
(C) Percentage of Gag p24$^+$ CD4 T cells after cultured alone or co-cultured with antibody-treated or untreated NK cells for 4 hours. The value of each data point is the average of 2-3 technical replicates. n=9.
(D) Percent reduction of the cell count of HIV-infected (Gag$^+$), Gag$^-$ bystanders and mock-infected CD4 T cells after cultured alone or co-cultured with antibody-treated or untreated NK cells for 4 hours. The value of each data point is the average of 2-3 technical replicates. n=9.
Statistical analysis in (C) and (D) was performed with the Wilcoxon signed-rank test.

Table S1: NK CyTOF panel

Isotope	NK Marker	Source	Clone	Panel
89Y	CD57	Biolegend	HCD57	Surface
112Cd/Qdot	CD19	Invitrogen	SJ25-C1	Surface
115In	CD3	Biolegend	UCHT1	Surface
141Pr	Granzyme B	Invitrogen	GB11	ICS
142Nd	MIP-1β	BD Biosciences	D21-1352	ICS
143Nd	NKG2C	R&D Systems	MAB1381	Surface
144Nd	CD161 (KLRB1)	BD Biosciences	DX12	Surface
145Nd	CD38	Biolegend	HIT2	Surface
146Nd	CD8	Biolegend	SK1	Surface
147Sm	CD107a (LAMP1)	Biolegend anti-APC	APC003	ICS
148Nd	LFA-1 (CD11a/CD18)	Biolegend	M24	Surface
149Sm	CD2 (LFA-2)	Biolegend	RPA-2.10	Surface
150Nd	HIV p24 core antigen	Abcam	39/5.4A	ICS
151Eu	Siglec-7	Biolegend	S7.7	Surface
152Sm	Perforin	Abcam	B-D48	ICS
153Eu	KIR2DS4 (CD158i)	R&D Systems	179315	Surface
154Sm	LILRB1 (ILT-2/CD85j)	R&D Systems	292319	Surface
155Gd	NKp46 (CD335)	Biolegend	9E2	Surface
156Gd	NKG2D	Biolegend	1D11	Surface
157Gd	TIGIT	R&D Systems	741182	Surface
158Gd	CD244 (2B4)	Biolegend	C1.7	Surface
159Tb	CD226 (DNAM-1)	BD Biosciences	DX11	Surface
160Gd	IFN-γ	BD Biosciences	B27	ICS
161Dy	NKp30 (CD337)	Biolegend	P30.15	Surface
162Dy	TNF-α	eBioscience	MAB11	ICS
163Dy	KIR3DL1	BD Biosciences	DX9	Surface
164Dy	NKp44	Biolegend	P44.8	Surface
165Ho	CD96 (TACTILE)	Biolegend	NK92.39	Surface
166Er	KIR2DL1	R&D Systems	143211	Surface
167Er	IP-10 (CXCL10)	Biolegend	J034D6	ICS
168Er	CD62L	Biolegend	DREG-56	Surface
169Tm	NKG2A	Fluidigm	Z199	Surface
170Er	KIR2DS2	Abcam	Polyclonal	Surface
171Yb	PD-1 (CD279)	Biolegend	EH12.2H7	Surface
172Yb	NTB-A	Biolegend	NT-7	Surface
174Yb	CD56	BD Pharmingen	NCAM16.2	Surface
175Lu	KIR2DL3	R&D Systems	180701	Surface
176Yb	CD69	Biolegend	FN50	Surface
209Bi	CD16	Fluidigm	3G8	Surface

Table S2: Ligand CyTOF panel

Isotope	NK Cell Ligand	Source	Clone	Panel
89Y	HLA-DR	Biolegend	L243	Surface
115In	CD3	Biolegend	UCHT1	Surface
143Nd	pan-HLA class I	Biolegend	W6/32	Surface
144Nd	CD7	Biolegend	CD7-6B7	Surface
145Nd	CD8	Biolegend	SK1	Surface
146Nd	CD48	Biolegend	BJ40	Surface
148Nd	ICAM1 (CD54)	Biolegend	HA58	Surface
149Sm	LLT-1	R&D	402659	Surface
150Nd	HIV p24 core antigen	Abcam	39/5.4A	ICS
151Eu	CD4	Biolegend	OKT4	Surface
153Eu	HLA-C	Millipore	DT9	Surface
154Sm	CCR2	Biolegend	K036C2	Surface
155Gd	HLA-E	Biolegend	3D12	Surface
156Gd	CD95	Biolegend	DX2	Surface
157Gd	Nectin-1 (CD111)	Biolegend	R1.302	Surface
158Gd	MICA	R&D	159227	Surface
158Gd	MICB	R&D	236511	Surface
159Tb	DR4 (CD261)	Biolegend	DJR1	Surface
159Tb	DR5 (CD262)	Biolegend	DJR2-2	Surface
161Dy	ULBP1	R&D	170818	Surface
161Dy	ULBP2 (5 and 6)	R&D	165903	Surface
164Dy	Nectin-2 (CD112)	Biolegend	TX31	Surface
165Ho	CD155 (PVR)	Biolegend	SKII.4	Surface
166Er	HLA-Bw4	Miltenyi	REA274	Surface
168Er	HLA-Bw6	Miltenyi	REA143	Surface
169Tm	CD14	Biolegend	M5E2	Surface
170Er	CD11b	Biolegend	ICRF44	Surface
171Yb	LFA-3 (CD58)	Biolegend	TS2/9	Surface
172Yb	CD33	Biolegend	WM53	Surface
174Yb	CD56	BD Pharmingen	NCAM16.2	Surface
176Yb	B7-H6	R&D	875001	Surface

Table S3: gRNAs used for CRISPR targeting

Target	Guide	crRNA Sequence
NCR3LG1 (B7-H6)	1#	mG*mG*GUGACCACCACCUCACAUGUUUUAGAGCUAUGCUGUUUUG
	2	mG*mA*GACACAAUGGCUCCAGGUGUUUUAGAGCUAUGCUGUUUUG
	3	mU*mC*ACGUCUAUGGGUAUCACCGUUUUAGAGCUAUGCUGUUUUG
CD48	1#	mU*mC*ACUUGGUACAUAUGACCGGUUUUAGAGCUAUGCUGUUUUG
	2	mA*mA*ACUGUCAUGUGUGAUACCGUUUUAGAGCUAUGCUGUUUUG
	3	mA*mU*GUACAGUGCGCCACUCUGGUUUUAGAGCUAUGCUGUUUUG
Nectin-2	1#	mG*mC*GAGUUCAAGUGCUACCCGGUUUUAGAGCUAUGCUGUUUUG
	2	mA*mC*ACUCACAGCGUACAGAGAGUUUUAGAGCUAUGCUGUUUUG
	3	mA*mG*CUGGGACCCAUCUUAGGGGUUUUAGAGCUAUGCUGUUUUG
CD58 (LFA-3)	1	mG*mA*GCAUUACAACAGCCAUCGGUUUUAGAGCUAUGCUGUUUUG
	2#	mG*mU*CAAUGCACAAGUUAGUGUGUUUUAGAGCUAUGCUGUUUUG
	3	mG*mC*AGCAGGCAGACCACGCUGGUUUUAGAGCUAUGCUGUUUUG
MICA	1#	mU*mG*UCCUGGGAAAUAAGACAUGUUUUAGAGCUAUGCUGUUUUG
	2	mU*mG*UUCUCCUCAGGACUACGCGUUUUAGAGCUAUGCUGUUUUG
	3	mA*mG*GUUAUAACGAAGACUGUGGUUUUAGAGCUAUGCUGUUUUG
MICB	1#	mG*mC*GAUAUCUGAAAUCCGGGGGUUUUAGAGCUAUGCUGUUUUG
	2	mA*mG*AAUGGGCAAGACCUCAGGGUUUUAGAGCUAUGCUGUUUUG
	3	mC*mA*UCCUGGGACAGCACCAUGGUUUUAGAGCUAUGCUGUUUUG
ULBP1	1#	mC*mC*UUGAACUUCACACCACUGGUUUUAGAGCUAUGCUGUUUUG
	2	mG*mC*AACUGCUUGACAUUCAAGGUUUUAGAGCUAUGCUGUUUUG
	3	mA*mG*UCAAUGUCACAAAAACCUGUUUUAGAGCUAUGCUGUUUUG
ULBP2	1#	mU*mC*UUCUCUGAGUCAAAGAGGGUUUUAGAGCUAUGCUGUUUUG
	2	mG*mC*UGGAGAAUUACACACCCAGUUUUAGAGCUAUGCUGUUUUG
	3	mA*mA*AGCUGAAGGACACAGCAGGUUUUAGAGCUAUGCUGUUUUG
AAVS1	1#	mG*mU*CACCAAUCCUGUCCCUAGGUUUUAGAGCUAUGCUGUUUUG

These single guide RNAs were used for editing CD4 T cells from donors 5 and 6 in Figures 4B, C, S8B, S9A and B and the donor in S8D. CD4 T cells from donors 1-4 in Figures 4B, C, S8A, S9A and B and the donor in S8C were edited with a pool of 3 guide RNAs as in the table for each NK cell ligand.
